# Freshness in Salmon by Hand-Held Devices: Methods in Feature Selection and Data Fusion for Spectroscopy

**DOI:** 10.1021/acsfoodscitech.4c00331

**Published:** 2024-08-22

**Authors:** Mike Hardy, Hossein Kashani Zadeh, Angelis Tzouchas, Fartash Vasefi, Nicholas MacKinnon, Gregory Bearman, Yaroslav Sokolov, Simon A. Haughey, Christopher T. Elliott

**Affiliations:** † National Measurement Laboratory: Centre of Excellence in Agriculture and Food Integrity, Institute for Global Food Security, School of Biological Sciences, Queen’s University Belfast, Belfast BT9 5DL, U.K.; ‡ SafetySpect Incorporated, Grand Forks, North Dakota 58202, United States; § Biomedical Engineering Program, University of North Dakota, Grand Forks, North Dakota 58202, United States

**Keywords:** fish freshness, food security, machine learning, data fusion, handheld spectroscopy

## Abstract

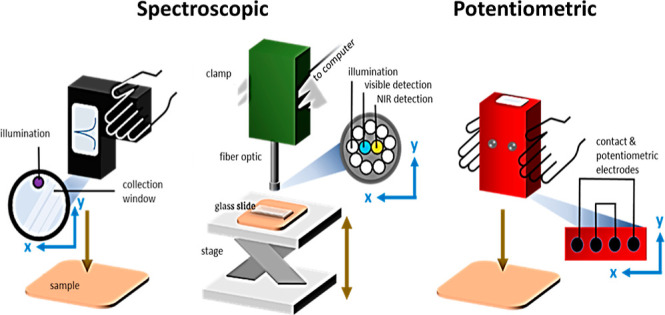

Salmon fillet was analyzed via hand-held optical devices: fluorescence (@340 nm) and absorption spectroscopy across the visible and near-infrared (NIR) range (400–1900 nm). Spectroscopic measurements were benchmarked with nucleotide assays and potentiometry in an exploratory set of experiments over 11 days, with changes to spectral profiles noted. A second enlarged spectroscopic data set, over a 17 day period, was then acquired, and fillet freshness was classified ±1 day via four machine learning (ML) algorithms: linear discriminant analysis, Gaussian naïve, weighted *K*-nearest neighbors, and an ensemble bagged tree method. Dual-mode data fusion returned almost perfect accuracies (mean = 99.5 ± 0.51%), while single-mode ML analyses (fluorescence, visible absorbance, and NIR absorbance) returned lower mean accuracies at greater spread (77.1 ± 10.1%). Single-mode fluorescence accuracy was especially poor; however, via principal component analysis, we found that a truncated fluorescence data set of four variables (wavelengths) could predict “fresh” and “spoilt” salmon fillet based on a subtle peak redshift as the fillet aged, albeit marginally short of statistical significance (95% confidence ellipse). Thus, whether by feature selection of one spectral data set, or the combination of multiple data sets through different modes, this study lays the foundation for better determination of fish freshness within the context of rapid spectroscopic analyses.

## 1 Introduction

Global food integrity, the assurance that consumer foodstuffs are safe to eat, of expected quality and authenticity continues to be of paramount concern,^1^ and increasingly complex supply chains have offered unease over food safety, quality, and outright food fraud.^2,3^ For instance, following the notable 2013 United Kingdom “horsemeat scandal”, an independent UK review recommended increased authenticity testing, the setting of threshold values for acceptable contamination, and better sharing of information during a crisis incident.^4^ Fish freshness is another prominent area of concern to food quality and potentially fraudulent activity where difficulty lies in determining the accurate time of fish post-mortem, except by protracted laboratory-based analyses.^5^ To this end, researchers have sought ways to determine seafood freshness more rapidly without decrease in accuracy, often with foci on device portability and cost-effectiveness.^6−13^

Sorak (2012) et al. identified the potential for hand-held vibrational spectroscopy [Fourier-transform infrared, near-infrared (NIR), Raman] in application-spaces other than their original design, homeland security.^14^ The move toward smaller instrumentation for analytical measurements has been driven by the industry need for devices that are inexpensive and can be used in the field with mitigation to reduction in performance and has been facilitated by progress in microelectro-mechanical systems and linear variable filters in a miniaturized NIR context.^15^ This is being supported by advancements in emerging lithographic techniques to manufacture tiny components reproducibly, inexpensively, and with high throughput.^16^ For instance, in a recent study on coriander adulteration, McVey (2021) et al. reported on the analytical performance of benchtop, portable and hand-held NIR instruments, demonstrating minimal performance deficit for smaller instrumentation.^17^ Similarly, Raman spectroscopy has been used with simple chemical reactions and plasmonics, the science of collective electron oscillations, to boost sensitivity,^18^ and surface enhanced Raman spectroscopy (SERS) studies can be viewed as quantitative.^19−23^ The technique has recently found use in foodstuff studies,^24^ for example, Ashley (2017) et al. quantitatively detected the antibiotic cloxacillin in porcine samples via gold nanopillar SERS and molecular imprinting to enhance analytical sensitivity and analytical specificity/selectivity, respectively.^25^ Development and proper characterization of novel SERS nanoplatforms across a wide range of application spaces is a continuing research interest.^26^

Another avenue to promote quantification is in advanced chemometric or machine learning (ML) analysis^14,27^ and has been recently discussed by Guo (2021) et al., who have set out a protocol for ML in a spectroscopy setting.^28^ Despite the emergence of complex data strategies appearing to be very modern, chemometrics have been employed within analytical sciences for rather quite some time,^29^ with their more recent proliferation signaling greater accessibility to scientists at large spurred by a buzz around *artificial intelligence*. There is a push for the adoption of ML in a variety of fields, including biological sciences,^30^ and recently, Feng (2021) et al. have reported on ML developments in foodstuff origin evaluation via hyperspectral imaging and visible and infrared absorbance spectroscopies.^31^ Recently, hyperspectral imaging, clustering techniques, and dimensionality reduction have been employed to investigate salmon freshness over a four-day time frame, concluding that incorporating high spatial resolution alongside spectral acquisition is useful for complete fillet freshness assessment.^32^ McGrath (2018) et al. have reviewed chemometrics and the movement toward identification of unknown adulterants via spectroscopy within food fraud.^33^ Advanced data techniques have been used across a wide range of foodstuffs, including in recent studies of cheese,^34^ milk,^35^ beef,^36^ and teas.^37^

Moreover, it may be that spectroscopy can detect matter which more sensitive techniques cannot, for instance. Hawkes (2019) et al. have reported organic compounds detectable by UV–visible range absorbance spectroscopy that are invisible to electrospray ionization mass spectrometry.^38^ Additionally, reference structures may not be available in a nontargeted analysis, and in this sense, matter has also been labeled “dark” to sensitive analytics. A de novo approach is possible, but is cumbersome, and only for known analytes, i.e., targeted analysis.^39^ Instead, vibrational spectroscopy, such as NIR absorbance, offers a “molecular fingerprint”, the spectra of which can be deduced via computational chemistry.

Despite the proliferation of spectroscopic analyses within food science and the adoption of statistical and ML strategies, robust models are still lacking. Data from different modalities are not routinely explored together. We propose that combined data sets provide a more accurate determination of produce freshness. This is an important application-space, and the deployment of more carefully considered data analysis schemes, such as spectral truncation and data fusion from different sources, is still not prevalent. Previously, we reported on using hyperspectral imaging technology to determine salmon freshness. Here, a maximum classification accuracy for freshness state over 4 days of 77% with a *K*-nearest neighbors algorithm was observed for point-spectra derived from the hyperspectral image. We noted the need to consider spatial nonuniformity in freshness state, i.e., differences in freshness across the fillet.^32^ Elsewhere, in a closely related study, we explored multiple measurements and averaging in fluorescence data for the determination of salmon fillet freshness, alongside ML methods.^40^ The UK-based arm of the study investigated fluorescence spectroscopy as a single analytical modality in detail, plotting spectral trends as the fish aged and noticing an increase in relative peak intensity ratio between the two salmon fluorescence peaks. A maximum classification accuracy (80% ± 1 day) for “fresh” vs “spoilt” salmon fillets with a support vector machine model was recorded. In the US-based arm of the study, a data fusion strategy for the three spectroscopic modes returned close to 100% accuracies for salmon. The best single-mode salmon classification accuracies were found from linear/quadratic discriminant analysis algorithms (LDA/QDA), which only showed minor deficit compared to fused data sets, often >90%.^40^ Reference (40) also contains an extensive background commentary on current methods within industry/research to determine state of fish freshness.

Herein, in the current study, which we term *Freshness in Salmon by Hand-held Devices* (“FiSH”), we extend our experiments to include more visible and NIR absorbance optical data and explore spectral feature selection as a tool to increase classification accuracy. We also benchmark results against the gold standard for rapid freshness determination, potentiometric analysis (the technique is detailed below), which was not performed previously. As a further benchmark, a greater number of nucleotide assays are performed to track fillet spoilage.

## 2 Materials and Methods

### 2.1 Sample Acquisition, Storage, and Preparation

The study herein is a two-phase collaboration among Queen’s University Belfast (QUB), UK, and SafetySpect Inc., ND, USA. The UK study (Phase 1) used two salmon fillets; the US study (Phase 2) used one salmon fillet (see Supporting Information Figure S1 for visual description of experiments and timeline). In Phase 1 of experiments, salmon fillet (“Fillet 1”) was purchased from a fresh seafood store in Kilkeel, County Down, Northern Ireland, and transported to the QUB ASSET laboratory, vacuum-packed. The salmon was farmed locally and slaughtered, and bones along the lateral line were removed (“pinning”) on the day of purchase. No further processing of the salmon was performed, apart from cutting the fillet. The fillet was refrigerated when not being analyzed at 4 °C (39 °F). An initial salmon fillet (“Fillet 0”) was purchased similarly 14 days previous and used for initial calibration measurements. Fillet 1 was divided into head and tail sections (Figure 2a, top), where the head was used for optical (flesh side only) and potentiometric measurements (skin side and flesh side) and the tail side was used for nucleotide assays (skin removed). Note, the cuts were taken from the most central regions of the tail section, i.e., closest to head section cut, for most meaningful comparison with optical measurements.

**Figure 1:**
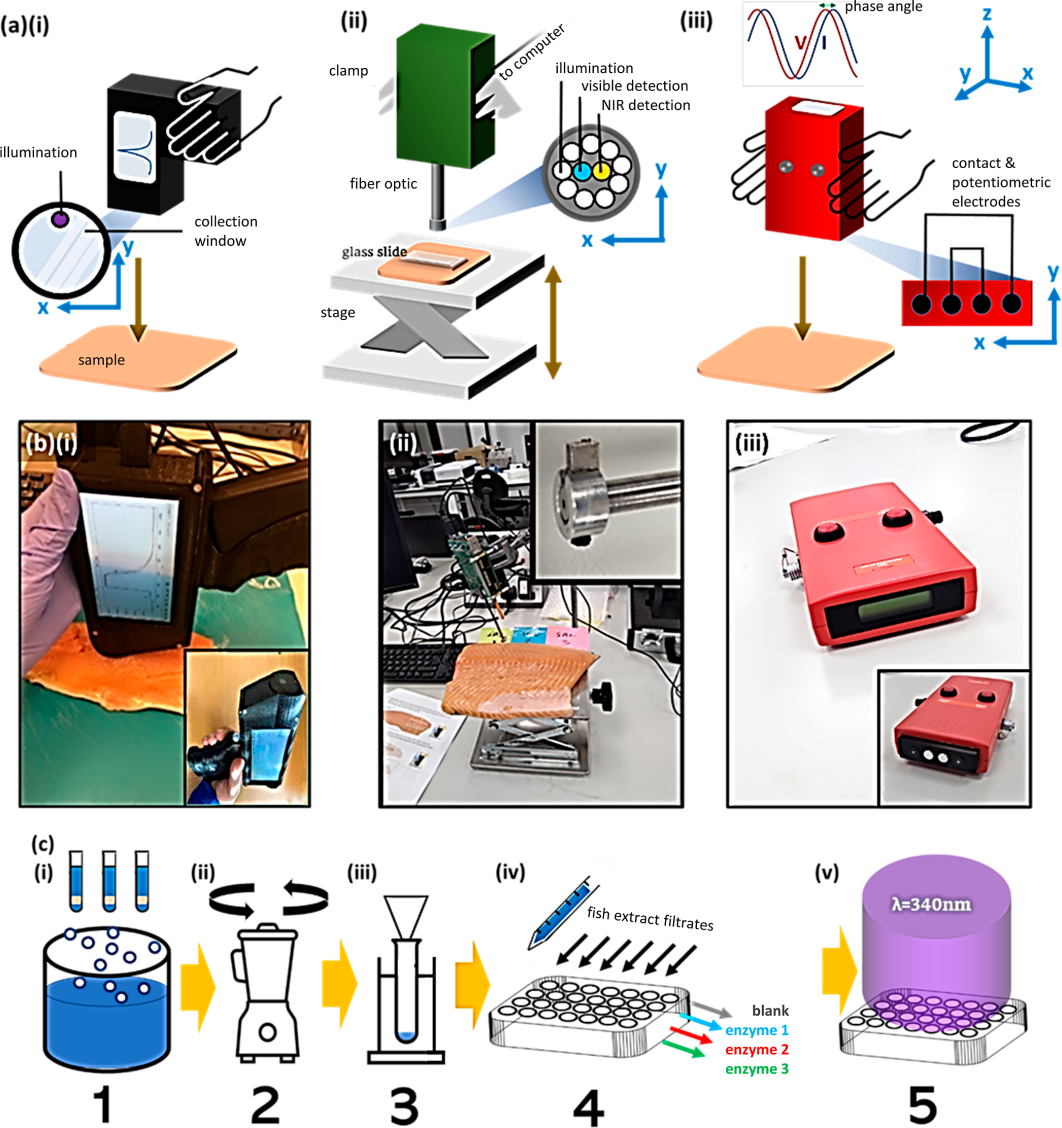
Overview of experimental techniques. (a) Schematics of (i) fluorescence, (ii) visible/NIR absorbance spectrometer devices, and (iii) potentiometric device for measuring electrical signals through specimens, with [b(i–iii)] accompanying photographs. (c) Nucleotide extraction procedure summary: (i) cooking fillet samples @ca. 100 °C (three replicates), (ii) blending/homogenizing, (iii) filtration, (iv) enzyme addition, and (v) UV light absorbance measurement with plate reader. Part [a(i)] adapted and reprinted with permission from ref (40) Copyright 2023 MDPI CC-BY-4.0 https://creativecommons.org/licenses/by/4.0/.

**Figure 2:**
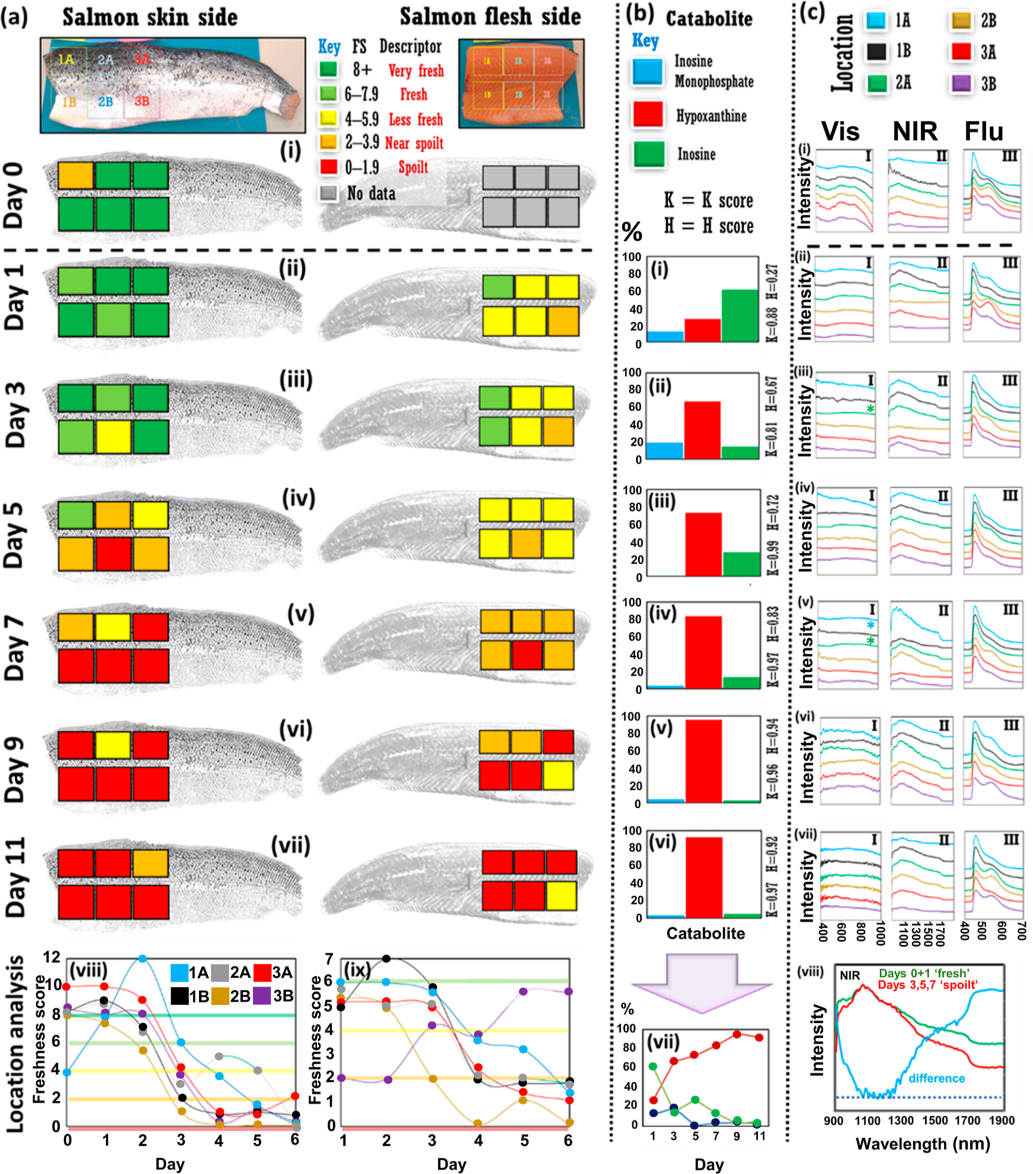
Phase 1 benchmarking/exploratory experiments on salmon fillet. (a) Potentiometric readings for 12 different fillet locations on fillet head region (6 skin side, 6 flesh side) for 11 day period (i–vii) with summaries for skin and flesh side in (viii,ix). Colored bars in (viii,ix) correspond to FS as in (a) key (top). (b) Relative nucleotide percentages for inosine monophosphate (blue bar), inosine (green bar), and hypoxanthine (red bar) over experiments (i–vi). *K*-Values and *H*-values indicated adjacent to the right-hand *y*-axis. Summary of variation in nucleotide relative percentages given in organoleptic chart in (vii). Please see Supporting Information Section S4: “Nucleotide Assay” for more details. (c) Optical data for six locations on fillet flesh side [as demarcated in (a)] over 11 experimental days (i–vii). Spectra, one/location, color-coded, key at (c) top. I, II, and III series for visible absorbance (400–1000 nm), NIR (900–1900 nm), and fluorescence (400–700 nm) data, respectively. Fluorescence spectra are truncated to the region of interest. All spectra are offset for clarity. All spectra acquired with glass slide spacer except those with adjacent asterisks (iii,v) for visible range spectra (I series) where artifactual features were observed. (viii) Pooled NIR spectra showing difference between mean of the six spectra Day0 + six spectra from Day1 “fresh” (green line) vs those from Days 3, 5, and 7 “spoilt” (red line). Note, power setting for absorbance spectra was 30% higher for baseline (Day0) measurements and optimized on subsequent days at lower intensity (color in print/online). For color-impaired and black and white readers, spectra in (c) are arranged 1A to 3B in order, top-to-bottom.

In Phase 2 of experiments, another salmon fillet (“Fillet 2”) was acquired at SafetySpect Inc. in the USA and more measurements taken for ML analysis. The salmon fillet was purchased from Fulton Fish Market, New York, USA, a reputable online vendor that ensured specimen traceability. The salmon was delivered frozen and placed into a −20 °C (−4 °F) freezer before transfer to a 4 °C (39 °F) fridge for 24 h before commencing experiments.

### 2.2 Spectroscopy Measurements: Visible/NIR Absorbance and Fluorescence

Phase 1 visible/NIR (vis/NIR) absorbance measurements (400–1900 nm) were taken through a glass slide to avoid occlusion of the light by pieces of fish flesh, especially pertinent past Day7 whenever the flesh became more viscous (with a surface texture resembling “wood glue” i.e. polyvinyl acetate). To note, the NIR region referred to here may also be designated “shortwave infrared” (SWIR). The terms can be used largely interchangeably; NIR covers a span from approximately 750–2500 nm, and SWIR an overlap of this range from around 1400–3000 nm, although exact definitions in the literature may differ slightly. A short protrusive collar (ca. 0.5 mm) was also used to protect the fiber-optic cable head [inset, Figure 1b(ii)]. Wavey spectral patterns due to interfacial interference within the glass slide (Fabry–Perot etaloning) were observed in some measurements, but this was not ubiquitous; an antireflective coating may be useful in subsequent device designs. The presence, or lack of appearance, of such optical interference fringes, appearing as undulations in the spectra, was assumed to be related to the alignment of the spectrometer fiber optic head with the slide and/or the quality of manufacture of the optical cavity (glass slide). Fluorescence measurements with excitation at 340 nm were taken with a hand-held fluorescence spectrometer. The measurement window was cleaned as necessary.

Across both experimental phases, at both research institutions, identical devices were used, provided by INSION GmbH (Obersulm–Willsbach, Baden-Wuerttemberg, Germany). Absorbance data were divided by a Spectralon reference measurement. Dark current was subtracted. Fluorescence spectra were not similarly treated, in line with the standard fluorescence procedure; this is viewed as unnecessary with variations less susceptible to external interference.

### 2.3 Potentiometric Measurements

Potentiometric (electrical) measurements were taken using the Distell Torrymeter (Distell Industries Ltd., Fauldhouse, West Lothian, Scotland, UK): five per measurement day, and a mean value calculated. The device is depressed into fish fillet, and a small current is passed through the sample measuring the resulting lag between the phase in the voltage and current signals (phase angle, complex impedance), related to the integrity of the fish cell membrane, which acts as a capacitor. The device measurement head was cleaned as required. The device head also includes additional two electrodes to monitor adequate sample contact [see Figure 1a(iii)]. Potentiometry via the Torrymeter device is evaluated with a *Freshness Score* (FS), which can be related to sensory, i.e., organoleptic descriptors, and explanations of the acceptability of the product to consumers.^41^ Repeatability of potentiometric measurements was assessed (Supporting Information, Figure S2). A limited set of organoleptic measurements was also conducted by independent observers alongside potentiometry (Supporting Information Section Figure S3). Further details are in Supporting Information Section S2.

### 2.4 Nucleotide Assay

Nucleotide extraction assays were performed alongside optical and potentiometric measurements as part of the Phase 1 experiments. The procedure involves the determination of the relative amounts of specific metabolic molecules, which, post-mortem, always degrade in one direction, i.e., catabolism. Thus, their relative quantities can give an indication of the fish freshness. The procedure is outlined in Figure 1c. Small portions (5 to 15 g) of fish were cut from the part of the fillet designated for the nucleotide assay measurements (three replicates), and distilled water was added and heated for 20 min at ca. 100 °C/212 °F. The fish was then homogenized and filtered. When the filtrate was cloudy, it was rectified by a subsequent centrifugation step. Enzymes were added to determine the respective amount of inosine, inosine monophosphate (IMP), and hypoxanthine (Hx) via the production of the metabolic coenzyme, nicotinamide adenine dinucleotide (NADH) (C_21_H_27_N_7_O_14_P_2_). The resulting well-plate was shaken at 600 rpm for 60 s before absorbance measurements were taken each minute over a period of up to 60 min to observe reaction kinetics. When IMP was observed to be negative, or Hx greater than 100%, typically at advanced fillet decay, values were set at 0 or 100. Mean values were taken from replicates (*n* = 3). *K*-value and *H*-values were calculated. The *K*- and *H*-values are metrics used to evaluate the freshness of fish products based on the relative percentages of the three catabolites (see eqs 1a and 1b). Full nucleotide assay procedure is available from NovoCIB SAS, Lyon, France 69003.^42^ Additional details are in Supporting Information Section S4.
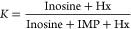
1a
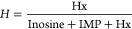
1b

### 2.5 Additional Experiments

**Figure 3:**
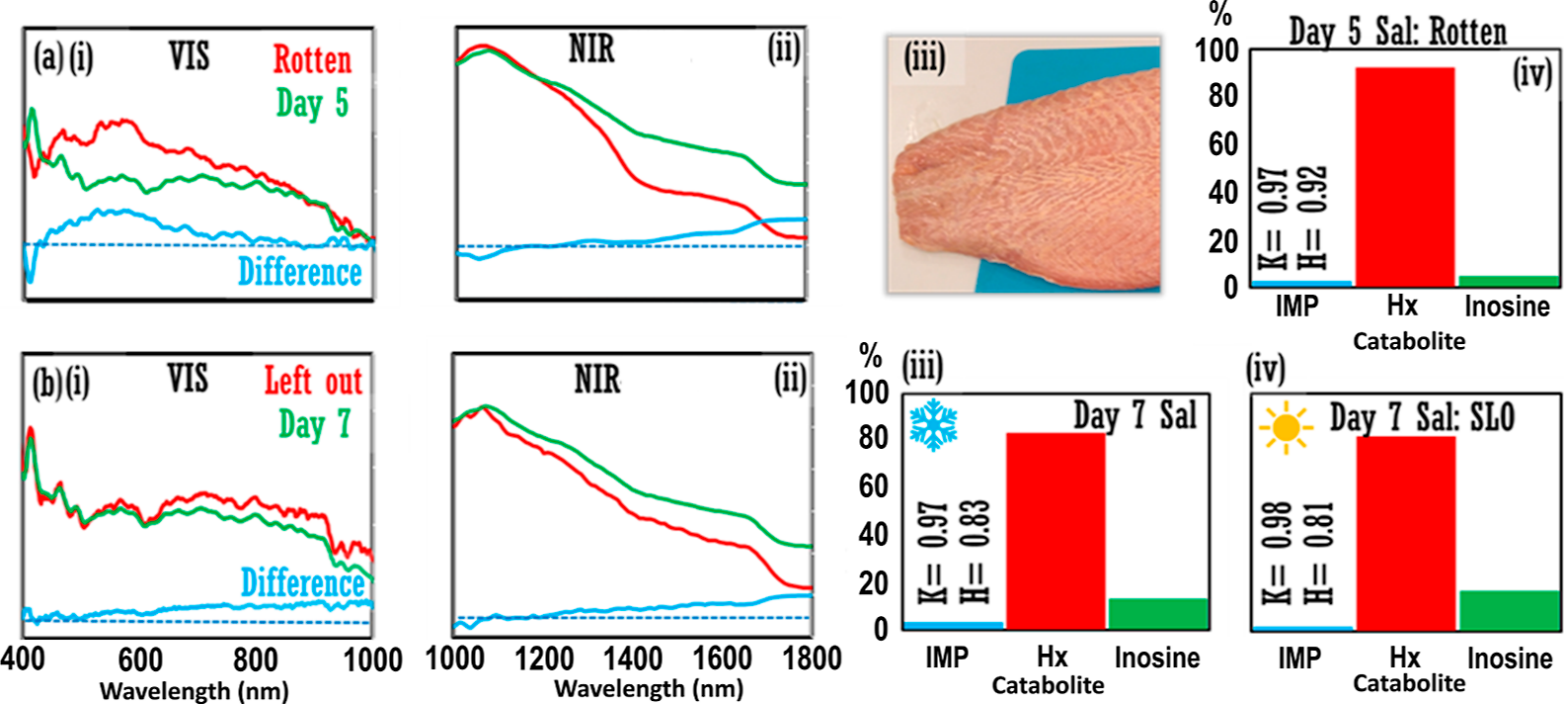
Additional experiments. (a) Comparison of Day5 salmon from Phase 1 experiment (“Fillet 1”, green line) and rotten salmon fillet (“Fillet 0”, red line) across (i) visible and (ii) NIR ranges. (iii) Photograph of rotten salmon fillet. Catabolite relative percentages performed on rotten salmon fillet, Fillet 0, on Day5. Catabolite data for Day5 salmon fillet, Fillet 1, are in Figure 2b(iii). (b) Comparison of Day7 salmon fillet, Fillet 1, which was refrigerated at 4 °C/39 °F when not being analyzed as normal (green line), and a cut of Fillet 1 that had been left out on the benchtop in the temperature-controlled laboratory for ca. 30 h previous, for (i) visible and (ii) NIR ranges. Catabolite relative percentages for Day7 salmon fillet (iii) refrigerated, and (iv) left out on lab bench (“SLO”). Inset: *K*-value and *H*-value. [b(iii)] is replotted from Figure 2b(iv) for easy comparison with SLO catabolite percentages in [b(iv)]. Light blue lines in [a(i,ii)] and [b(i,ii)] represent difference spectra; blue dash = unity position (color in print/online).

Two tangential experiments in Phase 1 were also performed. First, a putrid salmon fillet (“Fillet 0”), acquired similarly to the first fillet but purchased 14 days previous to the start of the main exploratory experiment and kept in refrigeration except when used for calibration measurements, was compared to Fillet 1 on Day5 (Figure 3). Second, a portion of Fillet 1 was cut off at ca. 22:00 on Day4 evening, covered, and left out on the lab bench in a temperature-controlled environment (ca. 25 °C/77 °F) until measurement on Day7 (ca. 30 h outside refrigeration), hereafter referred to as the “Salmon Left Out” sample (SLO). Optical spectra and nucleotide data were compared (see Figure 3).

### 2.6 Phase 2 Experiments

In experimental Phase 2, at the US-based lab, experiments were conducted as above with some variations. “Fillet 2” measurements were taken randomly spread across the fillet with a focus on obtaining a greater number of measurements for ML analysis. Data fusion was performed on the Fillet 2 salmon data set via concatenation of the following:

1. fluorescence and visible absorbance data,
2. fluorescence and IR absorbance data, and
3. visible absorbance and NIR absorbance data,

all via the method of unique pairing.

This method involves the random assignment (pairing) of data points from one data set to another. This is necessary where data sets have no obvious way to combine them, offering an effective technique to fuse analytical information from different devices/modalities. A summary of the experiments is depicted in Supporting Information Section Figure S1.

### 2.7 Data Analysis

Phase 1 data analysis was performed in R Studio, “prcomp” base R function was used for principal component analysis (PCA) and plotted via package: “ggplot2”.^43^ The R Kohonen package was used for self-organizing map (SOM) analysis and plotting.^44^ Otherwise, Microsoft Excel was used. Phase 2: Python SciKit Learn was used for LDA, weighted *K*-nearest neighbors (WKNN), Gaussian naïve (GN), and ensemble bagged tree (EBT) classification.^45^ The ML algorithms can be summarized: PCA is a well-known unsupervised dimensionality reduction technique that seeks to “redefine” axes to explain maximal variance in reconstituted variables known as principal components.^46^ PCA is often thus used as a foundational tool in ML for purposes of parsimony/computational tractability and the creation of more robust/rugged analytical models.^47^ PCA was used as an initial step for the Phase 2 study for feed into classifiers with an optimal number of PCs selected in each case. LDA is also a dimensionality reduction technique but supervised, i.e., class information is supplied, that minimizes scatter within classes and maximizes variance between classes with a linear decision boundary. GN is a Bayesian classifier that, by definition, incorporates prior probabilities of events into classification and that assumes data in the training data set is normally distributed. It is distinct from the Multinomial Naïve Bayes classification method, which considers discrete data sets. WKNN is a clustering algorithm that then matches the newly supplied data to the most proximal data (spectral angle calculation) already present, where the addendum of being “weighted” assigns greater importance to closer data points in multidimensional space, not considered in ordinary KNN models. SOM is an unsupervised artificial neural network, similar to clustering when used with low dimensional data, where “weight vectors”, which describe how nodes in the network are connected, are updated as data are continually added to the map. EBT is a supervised ML method where a collection of decision trees is used and a randomly selected, replaced subset of data is used to train each tree.

## 3 Results and Discussion

### 3.1 Phase 1 Results (UK)

Potentiometric measurements correlated with nucleotide assay measurements showing a general trend of decay. This is demonstrated by the increasing FS number output on the Torrymeter device display and the increase in percentages of the end-stage catabolites, Inosine, and Hx (and consequently *K*-value metric).^41^ According to potentiometric assessment (Figure 2a), the fillet was “fresh” (generally, FS > 6, skin side) and “spoilt” by the stage of Day5–Day7 (generally, FS < 4, skin side). Variation in FS in different regions was noted, notably Day5 skin side, where there were FSs across a significant part of the device range (0–8). A graphical summary of the FS for the different prescribed locations as a function of measurement day for skin and flesh fillet sides is plotted in Figure 2a(viii,ix).

Nucleotide relative percentages are plotted in Figure 2b(i–vi), *K*-values were calculated at 0.88 and 0.81 for Day1 and Day3 and >0.90 thereafter. A nucleotide plot [Figure 2b(vii)] shows the usual *IMP → INO → Hx* catabolic transition as the fillet ages [Figure 2b(vii)].

Spectroscopic measurements for visible absorbance (400–1000 nm), NIR absorbance (900–1900 nm), and fluorescence spectroscopy (excitation @340 nm) are plotted in Figure 2c for days 0–11 (i–vii) with the three different spectroscopic modalities plotted adjacent for easy comparison (I, II, III series). All spectra are on arbitrary intensity scales and are offset for clarity. Visible range spectra were noisy and conveyed only subtle spectra changes day to day. Contrariwise, NIR spectra displayed an increase in gradient across the spectra range evidenced by the 1100 nm-peak normalized mean spectra in Figure 2c(viii), i.e., the red “spoilt” spectrum falls off much more than the green “fresh” spectrum with regard to intensity at longer wavelengths. Fluorescence data varied greatly in spectral profile where a significant shoulder peak ca. 550 nm appeared erratically, sometimes as a distinct spectral feature [e.g., Day1, location 3A, red line in Figure 2c(ii)(III)], with no clear relationship to measurement day or fillet location upon cursory visual inspection.

Additional optical absorbance measurements on Day5 showed spectral differences between the Day5 fillet and a rotted salmon fillet [pictured, Figure 3a(iii)], for visible data and again for NIR, displaying a further increasing spectral gradient, as in Figure 2c. The increased decay of the rotted fillet was also evident by nucleotide analysis where *K* = 0.97 and *H* = 0.92 [Figure 3a(iv)], in comparison to *K* = 0.99 and *H* = 0.72 [Figure 2b(iii)] for Day5 fillet, which showed a higher inosine level. In the second tangential experiment, on Day7, SLO of refrigeration for 1 weekend period in a temperature-controlled laboratory was compared to the data for the Day7 Salmon fillet (refrigerated at 4 °C/39 °F as normal). The SLO sample did not show any noticeable color or olfactory difference relative to the salmon fillet kept in refrigeration, and comparable relative nucleotide percentages were obtained. Thus, a nucleotide assay could not discriminate between the SLO fillet and Day7 fillet (refrigerated *K* = 0.97, *H* = 0.83; SLO/nonrefrigerated *K* = 0.98, *H* = 0.81) [Figure 3b(iii,iv)]. However, spectral measurements demonstrated subtle changes across the full visible and NIR ranges [Figure 3b(i,ii)].

**Figure 4:**
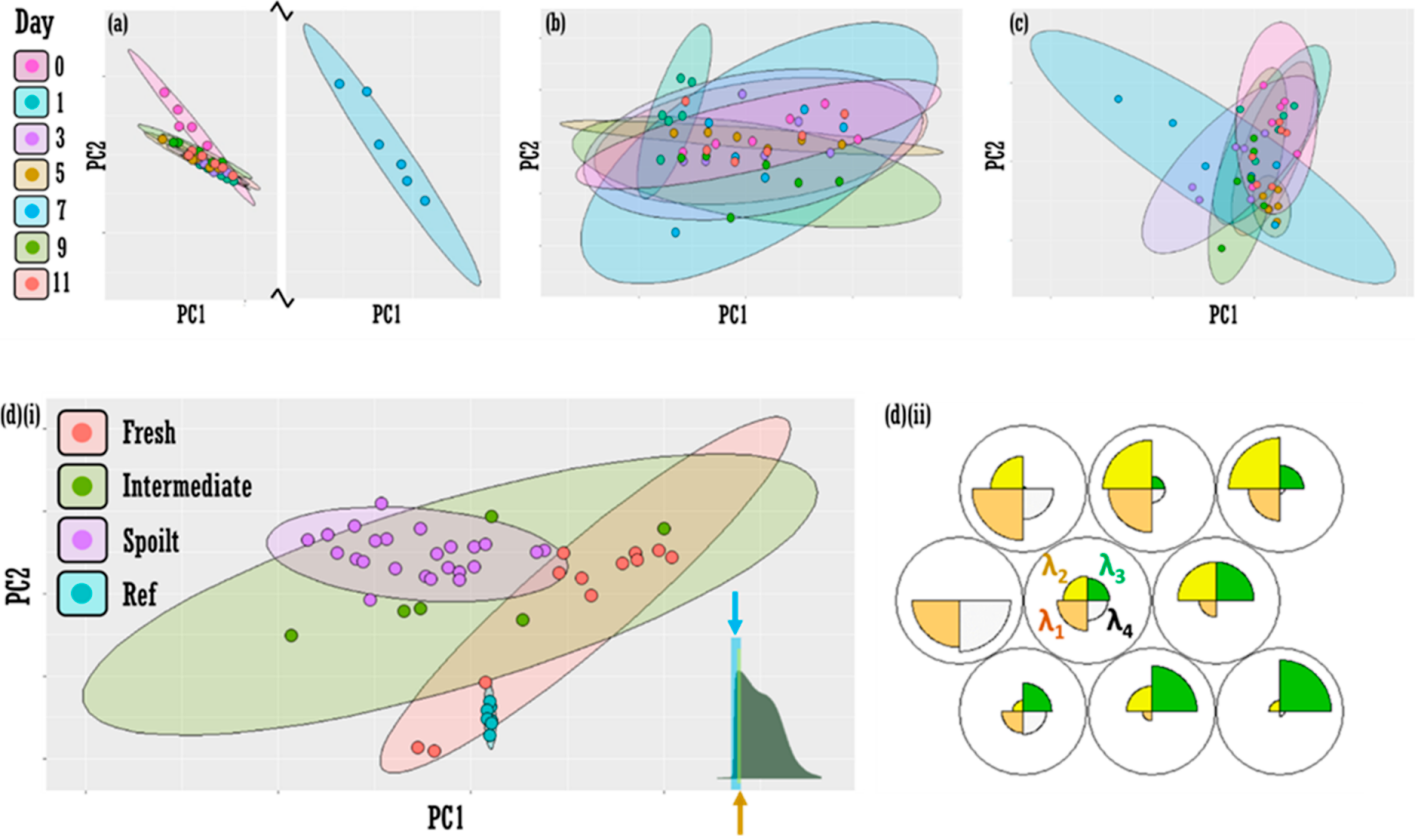
Feature selection as a method to improve fresh vs spoilt classification accuracy in fluorescence data. PCA (PC1 vs PC2) for (a) visible (b) NIR and (c) fluorescence data as a function of day. Confidence ellipses at 95%. [d(i)] Pooled data PCA plot (PC1 vs PC2) with feature selection showing discrimination between “fresh” (Days 0 and 1, orange ellipse) and “spoilt” (Days 5, 7, 9, and 11, purple ellipse). Day3 classes at “intermediate” (green ellipse). Nonpooled data PCA plot in the Supporting Information Section Figure S4. Features selected are wavelengths: 453, 455, 457, and 459 nm (to nearest integer value). Confidence ellipses set at 95%. Inset: yellow bar indicates approximate range of variables selected for truncated data set in [d(i)]. Expanded blue bar represents extended 13-variable model (435–459 nm), which provides poorer pooled class discrimination (see Supporting Information Section Figure S9). [d(ii)] Self-organizing maps “codes plot” displaying the map of the importance of each of the four variables (wavelengths: λ_1–4_ i.e., 453, 455, 457, and 459 nm) across all spectral data (color in print/online).

PCA was performed on the full spectroscopic data sets, visible (Figure 4a), NIR (Figure 4b), and fluorescence (Figure 4c) data sets. Classes (days) were statistically inseparable in all cases, as evidenced by overlapping 95% confidence ellipses. Feature selection of four variables (wavelengths) in the 453–459 nm spectral range for fluorescence data returns some significant separation (Supporting Information Figure S4), which can be visualized by pooled classes: designating Day0 and Day1 as “fresh”, Day3 as “intermediate” freshness, and days 5, 7, 9, and 11 as “spoilt” [orange, green, and purple data points, respectively, Figure 4d(i)]. The separation results from a small redshift in the low-wavelength fluorescence band ca. 450 nm (Supporting Information Figure S8) and can be evidenced by a SOM analysis, which displays a map of the most important variables (wavelengths) across the inputted (truncated) four-variable spectral data set [Figure 4d(ii)].

### 3.2 Phase 2 Results (USA)

**Figure 5:**
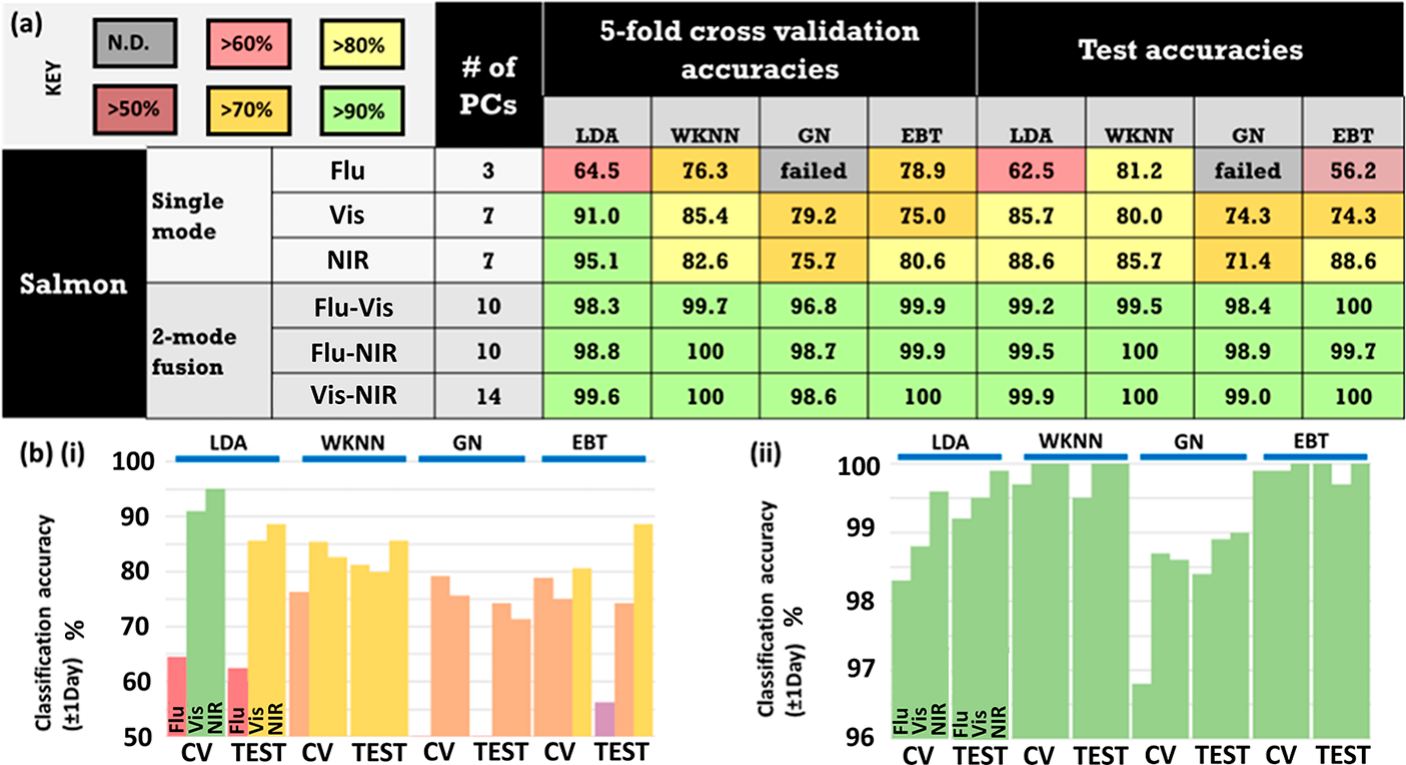
Phase 2 single-mode and dual-mode classification. (a) Salmon fillet classification accuracies ±1 day for 5-fold cross validation and test data sets for single-mode and dual-mode fusion for fluorescence (Flu), visible absorbance (vis), and NIR absorbance (NIR) phenomena, for four different classification algorithms: LDA, WKNN, Gaussian naïve (GN), and EBT. Accuracies color-coded: green: 90–100%; yellow: 80–89%; orange: 70–79%; red: 60–70%; brown 50–60%; and gray: no classification. “# of PCs” refers to the number of PCs retained for each classification model. An optimal number of PCs were selected for each model. Data were acquired from a second salmon fillet/set of experiments (Fillet 2, Phase 2). Data graphed in (b) for (i) single-mode and (ii) dual-mode. CV and test data are plotted for each algorithm (color in print/online).

In Phase 2, ML was performed on the second more extensive salmon data set in the form of LDA, WKNN, Gaussian naïve (GN), and EBT. 5-fold cross-validation (CV) and a test procedure were executed, and ±1 day accuracies were recorded for an optimal number of PCs for both single-mode and dual-mode data fusion (Figure 5). Single-mode data sets returned a mean overall classification accuracy of 78%, which improved to 99% with dual-mode fusion. NIR produced the highest single-mode accuracy at 84%, although the fluorescence GN models did not compute. All dual-fusion methods (Flu–vis, Flu–NIR, vis–NIR) were comparable, with recorded accuracies >96%. CV and test accuracies were comparable across all algorithms and whether single- or dual-mode was applied, bar an EBT model (CV-test accuracy ratio = 0.71), which was also markedly poor in absolute accuracies (CV: 79%; test: 56%). LDA, WKNN, GN, and EBT models returned mean classification accuracies for test data of 89.2% (S: 78.9%; F: 99.5%), 91.1% (S: 82.3%; F: 99.8%), 88.4% (S: 72.9%; F: 98.8%), and 86.4% (S: 73.0%; F: 99.9%), respectively, across fused (F) and nonfused/single-mode (S) data sets.

### 3.3 Discussion

Potentiometric measurements show different rates of spoilage across different fillet regions. In some cases, FS readings increase, e.g., Region 1A, skin side between Day1 and Day3 [Figure 2a(ii,iii)]. While this could be a result of device variation (see Supporting Information Figure S2), it may also be that there is some variation of freshness within each area. We note that some surface regions may have experienced some local fissure or have a higher microbacterial load, which may affect the rate of cell membrane integrity decay, i.e., the ability of the membrane to act capacitively. Similarly, nucleotide measurements from day-to-day were from proximal but nevertheless different fish cuts, which may have differing local nucleotide concentrations. Moreover, potentiometric and nucleotide measurements were from different fish segments, head, and tail (caudal). Tail regions tend to be thinner, and a greater surface-to-volume ratio would appear to speed up decay.^40^ The Torrymeter is intended for use on the skin-side and on the upper shoulder, just above the lateral (middle) line; however, our results suggest that it may be accurate in predicting decay for analysis elsewhere on the fillet and on the flesh (skin-off) side. This is useful because larger fish species may appear as cuts rather than full fillets and some species may have all skin removed. Both potentiometry and relative nucleotide analysis suggest that Day5–Day7 is the point of spoilage in our study where at Day5, the *K*-value is first >0.90 [Figure 2b(iii)], a cutoff value prescribed by Gopakumar (2002) as spoilt,^48^ or at Day7 most potentiometric FS readings are first <2 [Figure 2a(v)]. We note that the relatively low percentage of initial catabolite IMP at any stage suggests advancing decay at the purchase point, which is probably usual for shop-purchased fish given the rapid metabolism of IMP. *K*-Value can be as low as 0–20 in freshly slaughtered fish.^48^

Optical absorbance measurements, across the full visible–NIR i.e. 400–1900 nm range, are relatively featureless, barring some noise, e.g., Day11 <600 nm; >800 nm; differences are subtle. NIR absorbance spectral data were most promising as (an isolated) the spectral tool for fillet freshness determination, where a spectral gradient change was observed as the fillet aged [Figure 2c(viii)], albeit with low resolution (pooled days). Moreover, for NIR, some spectra patterns across locations are very uniform [Day5, Figure 2c(iv)(II)], others are much more erratic, e.g., Day7 [Figure 2c(v)(II series)], therefore averaging is still required. Day3 is classified as spoilt in NIR analysis due to the spectral similarity of Day3 with advancing days’ measurements and distinctness of the Day0 and Day1 NIR spectral profiles [Figure 2c(v)(II series)]. This may be as a result of using the flesh side, which may be decaying more rapidly than the fillet skin, which is supported by potentiometric measurements on the flesh side in Figure 2a. The comparison of the Day5 fillet with rotted salmon fillet in Figure 3 demonstrates that the gradient changes observed in the NIR spectra in the exploratory analysis (Figure 2c) may constitute meaningful changes corresponding to chemical/biological alteration to the fillet: the further increase in the gradient for the rotted salmon sample then represents an end point for the transition. In the second tangential experiment, the spectral differences observed in the absorbance data for the SLO sample vs Fillet 1 on Day7 [Figure 3b(i,ii)] provide evidence that spectroscopic means may be able to discern subtle biological/chemical changes that are not otherwise observable, even by nucleotide analyses [Figure 3b(iii,iv)].

While changes in the visible range (400 to 900 nm) across measurement days were minor in the Phase 1 data, the visible-range data improve classification accuracy in the dual-mode fusion analyses for Phase 2, and this suggests that these subtle variations may have some discriminatory power. Moreover, spectral differences in the visible range were evident in the comparisons with the rotting and SLO fillet samples. Likewise, fluorescence data also performed well as a fused data set (with NIR or visible absorbance) despite poor performance as an individual data set. This poor accuracy returned by single-mode fluorescence could be predicted from the erraticism of the spectral profile in the exploratory fluorescence measurements in Phase 1. We have addressed this variation previously, where we discuss intraclass variance with fluorescence spectral data and show the need for multiple fluorescence measurements for accurate freshness day classification.^40^

Feature selection is common in chemometric analyses. In early commentary, Wold (1995) lists feature selection as a use of PCA.^49^ Similarly, and commonly, discriminant analysis and genetic algorithms can be used to select variables among others, conferring the advantage of not only a reduced data load to input into a subsequent classifier but also more accurate models: only meaningful data are retained. Often features retained in spectroscopic data refer to variables (wavelengths) that correspond to entire peaks, i.e., peaks related to the target substance aside from other matrix constituents or interloping signatures otherwise. However, this does not abrogate partial peak selection, and herein, we observe a slight redshift in the fluorescence spectra as the salmon fillet ages, and thus, the isolation of a mere four variables around the escalation point of the cardinal fluorescence band (ca. 453–459 nm) provides discrimination of fresh and spoilt fillets. Interestingly, a modest increase in variables selected, to 13 (Supporting Information Figure S9), results in a loss of discriminatory power. We note that a four-variable model may not be optimal, and the inclusion of more wavelengths may capture the redshift more fully. The bunching of the reference data in Figure 4d(i) indicates that the changes are not artifactual, i.e., device-dependent.

The attraction in fluorescence feature selection is that single measurements might give a rough estimation of fillet freshness with a technology that is already well-established. Further, single-mode data are clearly cheaper and easier to work with than multimodal devices and data sets, and moreover, single-mode may be more accurate on external data; overfitting is less likely. Analogously, while broader spectral ranges incorporated in hand-held NIR devices at the cost of inferior signal-to-noise have been determined best for qualitative analyses, truncated spectral ranges and better signal-to-noise have proved optimal for quantitative determination.^15^ We observe that the high accuracies in fused data in our Phase 2 study may indicate overfitting, suggested by the high number of optimum PCs in the fused data models (10, 10, and 14). The high accuracy is also a result of a generous classification accuracy metric: ±1 day. A different strategy may be to examine all higher PCs closely and include only those showing class separation, regardless of optimum classification test accuracies for varying PC numbers (Supporting Information Figure S5). Further testing of externally acquired data will be necessary (external test data). Classification based on the exact day, especially over early days post-mortem, would be useful. Moreover, some ML models can be optimized, for example, by tuning the *k*-hyperparameter in KNN models, which corresponds to the number of proximal data points considered in class assignment for new data, or varying the KNN weighting kernel, which prescribes the function used to weight proximity for classification. Weighting has also been proposed in the context of improving Bayesian models.^50^ The nature of the decision boundary can also be altered in discriminant analysis, e.g., *QDA* and a common extension of EBTs are *random forests* where a subset of random features are also selected. We note that SOMs, like KNNs, when used as classifiers, may also be used in supervised fashion for classification, having recently appeared in the context of sugars identification,^51^ as well as outside of a foodstuff context in ocular constituent identification,^52^ and sex determination in saliva used as an auxiliary diagnostic.^53^

Data fusion can be viewed as another link in the chain when extracting useful information from big data^54^ but one that has not been used extensively within many recent food studies with spectroscopy.^33^ Selecting features and data fusion are compatible and can be described as “midlevel fusion” where elements of data sets are isolated before concatenation.^55^ Although cumbersome, midlevel fusion has been used successfully, and surprisingly, would appear more prominent than high-level fusion approaches for foodstuffs,^55^ although chemometric methods such as ensemble bagged trees (or random forests) are common classifiers in food studies and are tantamount to high-level fusion classification, albeit not designated as such.

A limitation in our study is the variation in salmon species used in both experimental phases; however, the nature of the UK–US collaboration meant exact salmon species usage was not possible. Second, and similarly, more data are preferable for more robust models. In practice, models may need to be updated regularly, but this may not be possible. There are a range of strategies to deal with model training from small data sets, for instance, shrinkage priors in the context of Bayesian penalisation.^56^ Third, indication of the exact time post-mortem would be useful but would require a commercial partner. This is possible in a further study. Estimation of the relative age of both Fillet 1 (Phase 1) and Fillet 2 (Phase 2) would have been beneficial, but the specialized nature of the nucleotide extraction assay limits this procedure, at present, to the UK laboratory. Despite the limitations of the current studies, we believe the current protocols can be adjusted to work for different species, meaning that accurate classifications of specimen freshness states can be determined for a wide range of seafood products. The current study provides evidence for the value of fusing data sets, selecting features from spectra, and multimodal spectroscopy, in salmon freshness classification, but this framework can be extended to other salmon and seafood species with adjustments, e.g., selecting different spectral features and different number of principal components for different fish types. These methods can also be used in fish species determination.^57^

In an initial exploratory set of experiments, salmon fillet was interrogated with optical means: fluorescence spectroscopy and absorbance spectroscopy over the visible and NIR range, and benchmarked against industry-standard potentiometry and the gold-standard laboratory procedure, nucleotide extraction assays, over a 11 day period. Variations in spectroscopic data as a function of measurement day were noted, with NIR absorbance demonstrating marked gradient change. Visible absorbance displayed more subtle spectral alterations and fluorescence, erratic peak profiles. Fluorescence data, however, showed promise for classification of fresh and spoilt salmon fillet when variables selected were limited in order to capture a redshift associated with sample decay. In the second set of experiments using identical hand-held optical devices, more data were acquired and ML methods employed with dual-mode data fusion to discern optimum classification algorithms and modalities. Single-mode analyses routinely returned classification accuracies ±1 day in the 70–90% across CV and test data; contrariwise, all fused methods returned accuracies >96%, demonstrating the potential predictive power of amalgamated data sets. A further study will be needed to ensure properly rugged models are developed. Our investigation paves the way for the development of portable devices for fish freshness measurement that balance speed and accuracy via the use of data analysis strategies whether by single or multimode spectroscopy.

## Data Availability

Data available on request due to privacy restrictions.

## Supporting Information Available

Experimental setup including the fundamental experimental design, electrical measurements, human sensory evaluations, nucleotide assay procedure, principal components analysis, overview of experiments, histogram for Torrymeter potentiometric measurements, organoleptic measurements, principal component analysis for salmon fillet fluorescence data for higher PCs, principal component analysis by fillet location, overlaid scaled fluorescence data, fluorescence redshift, and extended variable Principal Component Analysis plot (PDF)

## Notes

We declare that this research did not require ethics approval.

## Notes

All the authors of this manuscript consent to its publication.

## Notes

**Reuse**: Figure 1a(i),b(i) adapted from Kashani Zadeh et al. (2023) (ref (40)). CC-BY-4.0 https://creativecommons.org/licenses/by/4.0/#.

## References

[ref1] Galimberti A.; De Mattia F.; Losa A.; Bruni I.; Federici S.; Casiraghi M.; Martellos S.; Labra M. DNA Barcoding as a New Tool for Food Traceability. Food Res. Int. 2013, 50 (1), 55–63. 10.1016/j.foodres.2012.09.036.

[ref2] Fox M.; Mitchell M.; Dean M.; Elliott C.; Campbell K. The Seafood Supply Chain from a Fraudulent Perspective. Food Secur. 2018, 10 (4), 939–963. 10.1007/s12571-018-0826-z.

[ref3] Jia W.; van Ruth S.; Scollan N.; Koidis A. Hyperspectral Imaging (HSI) for Meat Quality Evaluation across the Supply Chain: Current and Future Trends. Curr. Res. Food Sci. 2022, 5, 1017–1027. 10.1016/j.crfs.2022.05.016. 35755306 PMC9218168

[ref4] Elliott C. Elliott Review into the Integrity and Assurance of Food Supply Networks—Final Report [Gov. Uk], 2014.

[ref5] Jones N. Hypoxanthine and Other Purine-Containing Fractions in Fish Muscle as Indices of Freshness. In The Technology of Fish Utilisation; Kreuzer R., Ed.; Fishing News Books Ltd.: London, 1965; pp 179–183.

[ref6] Cheng J.-H.; Sun D.-W. Hyperspectral Imaging as an Effective Tool for Quality Analysis and Control of Fish and Other Seafoods: Current Research and Potential Applications. Trends Food Sci. Technol. 2014, 37 (2), 78–91. 10.1016/j.tifs.2014.03.006.

[ref7] Karoui R.; Hassoun A.; Ethuin P. Front Face Fluorescence Spectroscopy Enables Rapid Differentiation of Fresh and Frozen-Thawed Sea Bass (Dicentrarchus Labrax) Fillets. J. Food Eng. 2017, 202, 89–98. 10.1016/j.jfoodeng.2017.01.018.

[ref8] Hassoun A.; Sahar A.; Lakhal L.; Aït-Kaddour A. Fluorescence Spectroscopy as a Rapid and Non-Destructive Method for Monitoring Quality and Authenticity of Fish and Meat Products: Impact of Different Preservation Conditions. LWT–Food Sci. Technol. 2019, 103 (December 2018), 279–292. 10.1016/j.lwt.2019.01.021.

[ref9] Hassoun A.; Karoui R. Front-Face Fluorescence Spectroscopy Coupled with Chemometric Tools for Monitoring Fish Freshness Stored under Different Refrigerated Conditions. Food Control 2015, 54, 240–249. 10.1016/j.foodcont.2015.01.042.

[ref10] ElMasry G.; Nakazawa N.; Okazaki E.; Nakauchi S. Non-Invasive Sensing of Freshness Indices of Frozen Fish and Fillets Using Pretreated Excitation-Emission Matrices. Sens. Actuators, B 2016, 228, 237–250. 10.1016/j.snb.2016.01.032.

[ref11] ElMasry G.; Nagai H.; Moria K.; Nakazawa N.; Tsuta M.; Sugiyama J.; Okazaki E.; Nakauchi S. Freshness Estimation of Intact Frozen Fish Using Fluorescence Spectroscopy and Chemometrics of Excitation-Emission Matrix. Talanta 2015, 143, 145–156. 10.1016/j.talanta.2015.05.031. 26078142

[ref12] Liao Q.; Suzuki T.; Yasushi K.; Al Riza D. F.; Kuramoto M.; Kondo N. Monitoring Red Sea Bream Scale Fluorescence as a Freshness Indicator. Fishes 2017, 2 (3), 10 10.3390/fishes2030010.

[ref13] Zhao Z. X.; Guo Y. P.; Wei J.; Chen Q. S.; Chen X. M. Fluorescent Copper Nanoclusters for Highly Sensitive Monitoring of Hypoxanthine in Fish. J. Anal Test 2021, 5 (1), 76–83. 10.1007/s41664-021-00166-z.

[ref14] Sorak D.; Herberholz L.; Iwascek S.; Altinpinar S.; Pfeifer F.; Sorak D.; Siesler H. W.; Iwascek S.; Altinpinar S. New Developments and Applications of Handheld Raman, Mid-Infrared, and Near-Infrared Spectrometers. Appl. Spectrosc. Rev. 2012, 47 (2), 83–115. 10.1080/05704928.2011.625748.

[ref15] Yan H.; Han B.; Siesler H. W. Handheld Near-Infrared Spectrometers: Reality and Empty Promises. Spectroscopy 2020, 35 (6), 15–18.

[ref16] Stokes K.; Clark K.; Odetade D.; Hardy M.; Goldberg Oppenheimer P. Advances in Lithographic Techniques for Precision Nanostructure Fabrication in Biomedical Applications. Discover Nano 2023, 18, 153 10.1186/s11671-023-03938-x. 38082047 PMC10713959

[ref17] McVey C.; Gordon U.; Haughey S. A.; Elliott C. T. Assessment of the Analytical Performance of Three Near-Infrared Spectroscopy Instruments (Benchtop, Handheld and Portable) through the Investigation of Coriander Seed Authenticity. Foods 2021, 10 (5), 956 10.3390/foods10050956. 33925477 PMC8145574

[ref18] Hardy M.; Doherty M. D.; Krstev I.; Maier K.; Möller T.; Müller G.; Dawson P. Detection of Low-Concentration Contaminants in Solution by Exploiting Chemical Derivatization in Surface-Enhanced Raman Spectroscopy. Anal. Chem. 2014, 86 (18), 9006–9012. 10.1021/ac5014095. 25133323

[ref19] Bell S. E. J.; Stewart A. Quantitative SERS Methods. In Surface Enhanced Raman Spectroscopy; Wiley VCH, 2010; pp 71–86.

[ref20] Fornasaro S.; Alsamad F.; Baia M.; Batista de Carvalho L. A. E.; Beleites C.; Byrne H. J.; Chiadò A.; Chis M.; Chisanga M.; Daniel A.; Dybas J.; Eppe G.; Falgayrac G.; Faulds K.; Gebavi H.; Giorgis F.; Goodacre R.; Graham D.; La Manna P.; Laing S.; Litti L.; Lyng F. M.; Malek K.; Malherbe C.; Marques M. P. M.; Meneghetti M.; Mitri E.; Mohaček-Grošev V.; Morasso C.; Muhamadali H.; Musto P.; Novara C.; Pannico M.; Penel G.; Piot O.; Rindzevicius T.; Rusu E. A.; Schmidt M. S.; Sergo V.; Sockalingum G. D.; Untereiner V.; Vanna R.; Wiercigroch E.; Bonifacio A. Surface Enhanced Raman Spectroscopy for Quantitative Analysis: Results of a Large-Scale European Multi-Instrument Interlaboratory Study. Anal. Chem. 2020, 92 (5), 4053–4064. 10.1021/acs.analchem.9b05658. 32045217 PMC7997108

[ref21] Yang J.; Palla M.; Bosco F. G.; Rindzevicius T.; Alstrøm T. S.; Schmidt M. S.; Boisen A.; Ju J.; Lin Q. Surface-Enhanced Raman Spectroscopy Based Quantitative Bioassay on Aptamer-Functionalized Nanopillars Using Large-Area Raman Mapping. ACS Nano 2013, 7 (6), 5350–5359. 10.1021/nn401199k. 23713574 PMC3915935

[ref22] Wei H.; McCarthy A.; Song J.; Zhou W.; Vikesland P. J. Quantitative SERS by Hot Spot Normalization - Surface Enhanced Rayleigh Band Intensity as an Alternative Evaluation Parameter for SERS Substrate Performance. Faraday Discuss. 2017, 205, 491–504. 10.1039/C7FD00125H. 28926064 PMC5709184

[ref23] Goodacre R.; Graham D.; Faulds K. Recent Developments in Quantitative SERS: Moving towards Absolute Quantification. Trends Anal. Chem. 2018, 102, 359–368. 10.1016/j.trac.2018.03.005.

[ref24] Procházka M. Bioanalytical SERS Applications. In Surface-Enhanced Raman Spectroscopy; Springer International Publishing, 2015; pp 61–91.

[ref25] Ashley J.; Wu K.; Hansen M. F.; Schmidt M. S.; Boisen A.; Sun Y. Quantitative Detection of Trace Level Cloxacillin in Food Samples Using Magnetic Molecularly Imprinted Polymer Extraction and Surface-Enhanced Raman Spectroscopy Nanopillars. Anal. Chem. 2017, 89 (21), 11484–11490. 10.1021/acs.analchem.7b02725. 28952718

[ref26] Hardy M.; Goldberg Oppenheimer P. When Is a Hotspot a Good Nanospot’ – Review of Analytical and Hotspot-Dominated Surface Enhanced Raman Spectroscopy Nanoplatforms. Nanoscale 2024, 16, 3293–3323. 10.1039/D3NR05332F. 38273798 PMC10868661

[ref27] Meza Ramirez C. A.; Greenop M.; Ashton L.; Rehman I. u. Applications of Machine Learning in Spectroscopy. Appl. Spectrosc. Rev. 2020, 56, 733–763. 10.1080/05704928.2020.1859525.

[ref28] Guo S.; Popp J.; Bocklitz T. Chemometric Analysis in Raman Spectroscopy from Experimental Design to Machine Learning–Based Modeling. Nat. Protoc. 2021, 16 (12), 5426–5459. 10.1038/s41596-021-00620-3. 34741152

[ref29] Handbook of Analytical Techniques; Günzler H., Williams A., Eds.; Wiley VCH: Weinheim, Germany, 2001; Vol. 1.

[ref30] Greener J.; Kandathil S. M.; Moffat L.; Jones D. T. A Guide to Machine Learning for Biologists. Nat. Rev. Mol. Cell Biol. 2022, 23 (1), 40–55. 10.1038/s41580-021-00407-0. 34518686

[ref31] Feng L.; Wu B.; Zhu S.; He Y.; Zhang C. Application of Visible/Infrared Spectroscopy and Hyperspectral Imaging With Machine Learning Techniques for Identifying Food Varieties and Geographical Origins. Front. Nutr. 2021, 8 (June), 680357 10.3389/fnut.2021.680357. 34222304 PMC8247466

[ref32] Hardy M.; Moser B.; Haughey S. A.; Elliott C. T. Does the Fish Rot from the Head? Hyperspectral Imaging and Machine Learning for the Evaluation of Fish Freshness. Chemom. Intell. Lab. Syst. 2024, 245, 105059 10.1016/j.chemolab.2023.105059.

[ref33] McGrath T. F.; Haughey S. A.; Patterson J.; Fauhl-Hassek C.; Donarski J.; Alewijn M.; van Ruth S.; Elliott C. T. What Are the Scientific Challenges in Moving from Targeted to Non-Targeted Methods for Food Fraud Testing and How Can They Be Addressed? – Spectroscopy Case Study. Trends Food Sci. Technol. 2018, 76 (April), 38–55. 10.1016/j.tifs.2018.04.001.

[ref34] Tarapoulouzi M.; Logan N.; Hardy M.; Montgomery H.; Haughey S. A.; Elliott C. T.; Theocharis C. R. A Pre-Trial Study to Identify Species of Origin in Halloumi Cheese Utilising Chemometrics with Near-Infrared and Hyperspectral Imaging Technologies. Analytica 2024, 5 (1), 17–27. 10.3390/analytica5010002.

[ref35] Tarapoulouzi M.; Kokkinofta R.; Theocharis C. R. Chemometric Analysis Combined with FTIR Spectroscopy of Milk and Halloumi Cheese Samples According to Species’ Origin. Food Sci. Nutr. 2020, 8, 3262–3273. 10.1002/fsn3.1603. 32724591 PMC7382104

[ref36] Haughey S. A.; Montgomery H.; Moser B.; Logan N.; Elliott C. T. Utilization of Hyperspectral Imaging with Chemometrics to Assess Beef Maturity. Foods 2023, 12 (24), 4500 10.3390/foods12244500. 38137302 PMC10743197

[ref37] Li Y.; Logan N.; Quinn B.; Hong Y.; Birse N.; Zhu H.; Haughey S.; Elliott C. T.; Wu D. Fingerprinting Black Tea: When Spectroscopy Meets Machine Learning a Novel Workflow for Geographical Origin Identification. Food Chem. 2024, 438, 138029 10.1016/j.foodchem.2023.138029. 38006696

[ref38] Hawkes J. A.; Sjöberg P. J. R.; Bergquist J.; Tranvik L. Complexity of Dissolved Organic Matter in the Molecular Size Dimension: Insights from Coupled Size Exclusion Chromatography Electrospray Ionisation Mass Spectrometry. Faraday Discuss. 2019, 218, 52–71. 10.1039/C8FD00222C. 31120465

[ref39] da Silva R. R.; Dorrestein P. C.; Quinn R. A. Illuminating the Dark Matter in Metabolomics. Proc. Natl. Acad. Sci. U.S.A. 2015, 112 (41), 12549–12550. 10.1073/pnas.1516878112. 26430243 PMC4611607

[ref40] Kashani Zadeh H.; Hardy M.; Sueker M.; Li Y.; Tzouchas A.; MacKinnon N.; Bearman G.; Haughey S. A.; Akhbardeh A.; Baek I.; Hwang C.; Qin J.; Tabb A. M.; Hellberg R. S.; Ismail S.; Reza H.; Vasefi F.; Kim M.; Tavakolian K.; Elliott C. T. Rapid Assessment of Fish Freshness for Multiple Supply-Chain Nodes Using Multi-Mode Spectroscopy and Fusion-Based Artificial Intelligence. Sensors 2023, 23 (11), 5149 10.3390/s23115149. 37299875 PMC10255221

[ref41] Organoleptic Charts. In Distell Torrymeter User Manual; Distell Industries Ltd.: Fauldhouse, West Lothian, UK, 2010.

[ref42] Balakireva L. PRECICE Nucleotides Assay Kits; NovoCIB. https://www.novocib.com/freshness-assay-kits.

[ref43] Wickham H.; Chang W.; Henry L.; Pedersen T. L.; Takahashi K.; Claus W.; Woo K.; Yutani H.; Dunnington D. Ggplot2: Elegant Graphics for Data Analysis; Springer-Verlag: New York, 2016.

[ref44] Wehrens R.; Buydens L. Self- and Super-Organizing Maps in R: The Kohonen Package. J. Stat. Software 2007, 21 (5), 1–19. 10.18637/jss.v021.i05.

[ref45] Pedregosa F.; Varoquaux G.; Gramfort A.; Michel V.; Thirion B.; Grisel O.; Blondel M.; Prettenhofer P.; Weiss R.; Dubourg V.; Vanderplas J.; Passos A.; Cournapeau D.; Brucher M.; Perrot M.; Duchesnay E. Scikit-Learn: Machine Learning in Python. J. Mach. Learn. Res. 2011, 12, 2825–2830.

[ref46] Beattie J. R.; Esmonde-White F. W. L. Exploration of Principal Component Analysis: Deriving Principal Component Analysis Visually Using Spectra. Appl. Spectrosc. 2021, 75 (4), 361–375. 10.1177/0003702820987847. 33393349

[ref47] Wold S. Chemometrics; What Do We Mean with It, and What Do We Want from It?. Chemom. Intell. Lab. Syst. 1995, 30, 109–115. 10.1016/0169-7439(95)00042-9.

[ref48] Gopakumar K. Textbook of Fish Processing Technology; Indian Council of Agricultural Research: New Dehli, 2002.

[ref49] Wold S. Chemometrics; What Do We Mean with It, and What Do We Want from It?. Chemom. Intell. Lab. Syst. 1995, 30, 109–115. 10.1016/0169-7439(95)00042-9.

[ref50] Rennie J. D. M.; Shih L.; Teevan J.; Karger D. R. Tackling the Poor Assumptions of Naive Bayes Text Classifiers. In Proceedings of the Twentieth International Conference on Machine Learning (ICML-2003), 2003; p 1973.

[ref51] De Carvalho Gomes P.; Hardy M.; Tagger Y.; Rickard J. J. S.; Mendes P.; Oppenheimer P. G. Optimization of Nanosubstrates toward Molecularly Surface-Functionalized Raman Spectroscopy. J. Phys. Chem. C 2022, 126, 13774–13784. 10.1021/acs.jpcc.2c03524. PMC9393890 36017358

[ref52] Banbury C.; Mason R.; Styles I.; Eisenstein N.; Clancy M.; Belli A.; Logan A.; Goldberg Oppenheimer P. Development of the Self Optimising Kohonen Index Network (SKiNET) for Raman Spectroscopy Based Detection of Anatomical Eye Tissue. Sci. Rep. 2019, 9 (1), 10812 10.1038/s41598-019-47205-5. 31346227 PMC6658481

[ref53] Buchan E.; Kelleher L.; Clancy M.; Stanley Rickard J. J.; Oppenheimer P. G. Spectroscopic Molecular-Fingerprint Profiling of Saliva. Anal. Chim. Acta 2021, 1185, 339074 10.1016/j.aca.2021.339074. 34711319

[ref54] Meng T.; Jing X.; Yan Z.; Pedrycz W. A Survey on Machine Learning for Data Fusion. Inf. Fusion 2020, 57 (2), 115–129. 10.1016/j.inffus.2019.12.001.

[ref55] Borràs E.; Ferré J.; Boqué R.; Mestres M.; Aceña L.; Busto O. Data Fusion Methodologies for Food and Beverage Authentication and Quality Assessment - A Review. Anal. Chim. Acta 2015, 891, 1–14. 10.1016/j.aca.2015.04.042. 26388360

[ref56] Chu H. O.; Buchan E.; Smith D.; Goldberg Oppenheimer P. Development and Application of an Optimised Bayesian Shrinkage Prior for Spectroscopic Biomedical Diagnostics. Comput. Methods Programs Biomed. 2024, 245, 108014 10.1016/j.cmpb.2024.108014. 38246097

[ref57] Sueker M.; Daghighi A.; Akhbardeh A.; MacKinnon N.; Bearman G.; Baek I.; Hwang C.; Qin J.; Tabb A. M.; Roungchun J. B.; Hellberg R. S.; Vasefi F.; Kim M.; Tavakolian K.; Kashani Zadeh H. A Novel Machine-Learning Framework Based on a Hierarchy of Dispute Models for the Identification of Fish Species Using Multi-Mode Spectroscopy. Sensors 2023, 23 (22), 9062 10.3390/s23229062. 38005450 PMC10674920

