# Supplementary material for: Freshness in Salmon by Hand-Held Devices: Methods in Feature Selection and Data Fusion for Spectroscopy
Source: ACS Food Sci Technol. 2024 Aug 22;4(12):2813–23. doi: 10.1021/acsfoodscitech.4c00331 (PMC11667728; doi:10.1021/acsfoodscitech.4c00331)
Supplement: Supplementary file 1 — fs4c00331_si_001.pdf [file fs4c00331_si_001.pdf]

–Supplementary Info–

Freshness in Salmon by Handheld Devices (FiSH): Methods in Feature Selection and Data Fusion for Spectroscopy

Mike Hardy[1,,a], Hossein Kashani Zadeh[2,3], Angelis Tzouchas[2], Fartash Vasefi[2], Nicholas MacKinnon[2], Gregory Bearman[2], Yaroslav Sokolov[2], Simon A. Haughey[1], Christopher T. Elliott[1].*

1. National Measurement Laboratory: Centre of Excellence in Agriculture and Food Integrity, Institute for Global Food Security, School of Biological Sciences, Queen's University Belfast, Belfast BT9 5DL, UK.
2. SafetySpect Inc., Grand Forks, ND 58202, USA.
3. Biomedical Engineering Program, University of North Dakota, Grand Forks, ND 58202, USA.

* Current address: Smart Nano NI, Centre for Quantum Materials and Technologies, School of Mathematics and Physics, Queen's University Belfast, Belfast BT7 1NN, UK.

a Corresponding author (mhardy04@qub.ac.uk)

S1. Experimental design

The experimental design (FigureS1) consisted of measurements with the three spectral modes (two devices), namely, visible absorbance, near-infrared, and fluorescence spectroscopy, on alternate days, from Day1–Day11. Nucleotide assay and potentiometric measurements were acquired concurrently. We note that comparison between fillets F1 and F2 would have been beneficial. We would have liked to have done a nucleotide assay procedure in the US laboratory in addition for cross-comparison, but we did not have the equipment to do so.

Regarding time between fillet purchase and measurements. The UK fillet was purchased in Kilkeel – the seafood capital of Northern Ireland, in the morning (~09:00) – and transported (vacuum-packed) to the Queen's University Belfast lab in Belfast and immediately put into refrigeration in early afternoon (~12:00). Time constraints meant that only potentiometric measurements were taken on 'Day 0' (afternoon/evening). A more local supplier would be beneficial.

On all other occasions, potentiometric/spectroscopic measurements were taken in the afternoon on alternative days and nucleotide assays performed late afternoon running into the evenings.

S2. Electrical measurements (potentiometry)

The performance of the potentiometric device was characterized through repeated measurements (FigureS2), where greater reproducibility was observed on the fillet skin side, which is prescribed as optimum for measurements (top shoulder, above lateral line). Measurements on the fillet flesh side appear much more scattered in histogram profile (FigureS2), however, the regional trends of decay were ‘sensible’ for flesh side measurements in the main study (Figure2(a), main text). This raises a question about repeated measurements in the calibration causing erroneous readings due to damage caused by repeated device depression into the fillet. Skin side may be more robust and less susceptible to damage from repeated measurement. Note, the Torrymeter guidelines state a warm-up device time of one minute. This was not timed in our experiments and may be worthwhile being observed strictly in future investigation.

S3. Human sensory evaluation (organoleptic measurements)

Organoleptic measurements were performed by independent observers, two per day, who were not informed of others’ evaluations or the time of purchase of the fillet (FigureS3). Participants were provided with sensory descriptors from the Distell organoleptic charts with corresponding freshness score. The respondents generally agreed on the freshness state of the fillet, barring the response of Respondent #2 on Day7 (FS=14). Evaluation was not possible in Day5 (weekend) and not useful on Day9 and Day11 whenever the fillet was evidently spoilt.

S4. Nucleotide Assay

Full details on nucleotide assay are available from NovoCIB (novocib.com). The nucleotide assay samples were stored at -20°C bar one complete defrost of all samples prior to experiments, after which they were re-frozen. On respective experimental days, each kit was then defrosted individually prior to measurement. Assay constituents were left to defrost on day of assay and performed in afternoon/evening. NovoCIB guidelines state that lyophilized enzymes, once hydrated, can be used more than once as long as stored correctly (up to three months), however, we did not do this, instead taking a new assay kit for each set of measurements (assay day). Assay reuse could be done in future for more economical practice. The nucleotide assay shows the general, expected, trend of catabolism – the transition from early-stage catabolites to late-stage catabolites post-mortem. This can be compared to calibration charts in the literature and elsewhere, however, for most meaningful comparison, the same type of fish (farmed Irish salmon) should be monitored in the same laboratory with more strictly monitored conditions (and used as a reference/calibration).

After enzymes were applied to fish filtrate and, well-plate was shaken, the reaction kinetics were closely monitored and read-off time varied according to whenever the plateau in absorbance was reached. Read-off time varied from 10 minutes to 60 minutes.

We note that significant variation in the three replicates (R1, R2, R3) often exists. For instance, in the Day3 measurements R1 and R3 returned inosine relative percentages of ~20% while R2 did not show the presence of inosine (13% IMP, 88% Hx; rounded). This could be as a result of genuine local variations in sample composition, or variation in assay performance. All such measurements were caried out by the same researcher. If one of the three replicates failed (noted below in TableS1). This data, then, was not included in subsequent catabolite calculations (if optical density, OD, was actually measured).

Note, ‘Day11’ (vi) nucleotide data was recorded on Day12 due to experimental constraints. Similarly, ‘Day1’ sample was frozen on Day0 upon arrival to the lab and dethawed on Day12 and assay performed to time-constraints (Figure2, main text).

We define the linearity of our plate reader (TECAN Sapphire 2) in the range <1.8 optical density (OD). Most final enzyme measurements (inosine) were within this range, except for a few exceptions, which were a little above 1.8, as listed below in TableS1). Many inosine monophosphate (IMP) OD measurements were low, indicative of small amounts of this early stage catabolite present, thus it is hard to quantify it accurately, falling below the quantification threshold for the plate reader.

Linear range for platereader defined as <1.8OD. Source=Novocib
Quantification threshold, typically >5% for any one catabolite. Source=Novocib
Catabolite Optical Densities (selected results which were low/high) thus affecting precise quantification. High values highlighted in red.
**Day 1** (n=2) IMP R1= 0.28, R3=0.73
Hx R1=0.59, R3=0.75
Ino R1=1.16, R3=2.44
**Day 3** (n=3) IMP R1=0.46, R2=0.35, R3=0.40
Hx R1=0.66, R2=0.72, R3=0.65
Ino= R1=0.72, R2=0.72, R3=0.76
**Day 5** (n=2) IMP R1=0.27, R2=0.25
**Day 7** (n=3) IMP R1=0.32, R2=0.34, R3=0.32
(n=3) Hx R1= 2.05, R2=2.17, R3=2.05
(n=3) Ino R1=2.28, R2=2.49, R3=2.34
**Day 9** (n=3) IMP R1=0.41, R2=0.25, R3=0.31
**Day 11** (n=2) IMP R1=0.35, R2=0.34

Table S1. Selected catabolite optical densities, which were low or high, thus affecting precise quantification.

S5. Principal Component Analysis

Principal Component Analysis (PCA) plot (PC1 vs PC2) for the truncated four-variable fluorescence model is presented in FigureS4, analogous to main text Figure4(d)(i) but here classified with individual days, rather than pooled descriptors of 'fresh', 'intermediate' and 'spoilt'. No significant groupings were observed for fluorescence day-classified higher PC plots (FigureS5). Moreover, PCs grouped based on location for fluorescence data showed no separation indicating that differences between the six different head locations was not significant in our study (FigureS6). As indicated in main text body, single fluorescence measurements provide no indication of freshness; the spectral profiles are erratic, as evidenced in overlaid spectral plots for the different locations for each day in FigureS7 (replotted from main body Figure2(c), III series). We have discussed the varying intensity of the second fluorescence band, and the need for significant averaging of fluorescence measurements in the context of fish freshness evaluation previously elsewhere[1]. As discussed in the main text body herein, another option is feature section, owing to a small redshift in the first fluorescence peak; zoomed-in spectral profiles as a function of day are presented in FigureS8. We have also noted that our four-variable PCA model may be sub-optimal and that the inclusion of more variables (wavelengths) may produce better fresh-spoilt separation. A 13-variable PCA model with pooled days for fluorescence data is presented in FigureS9.

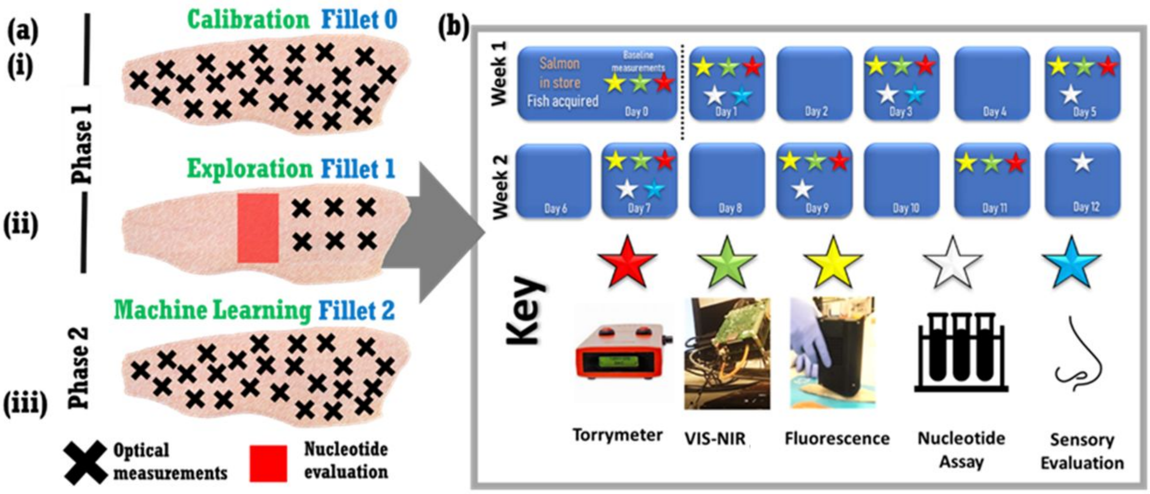

Fig.S1 *Overview of experiments (a) Experimental design (b) Phase 1 outline of procedures by measurement Day.*

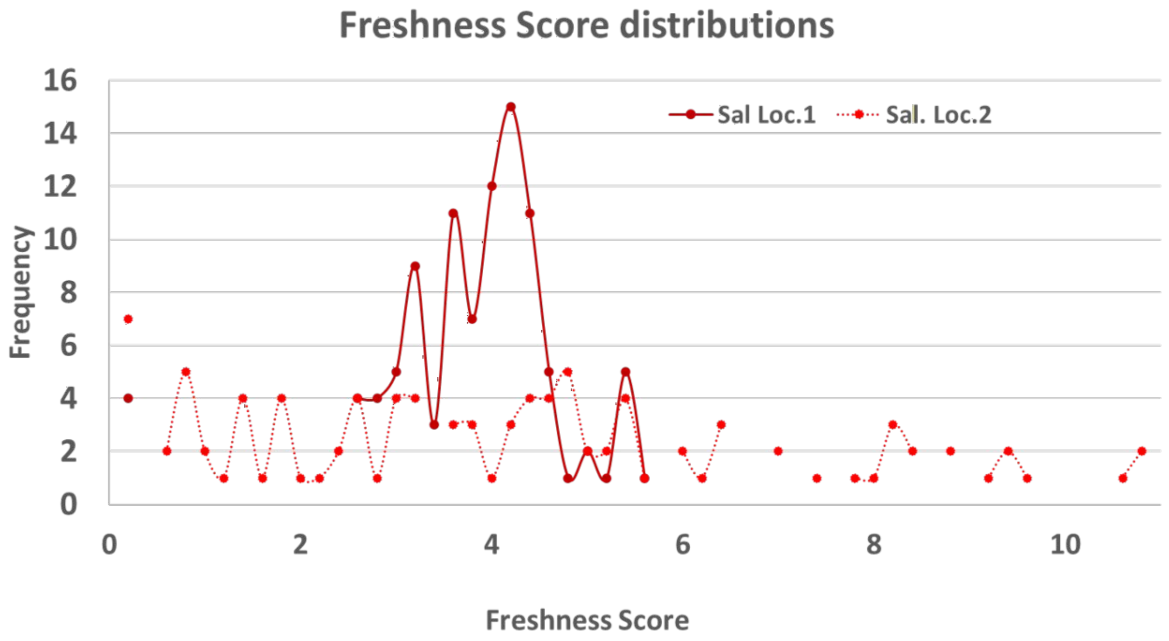

Fig.S2 *Histogram for Torrymeter potentiometric measurements for two different fillet locations (a) Location 1 (skin side) (b) Location 2 (flesh side). Measurements on fresh salmon fillet ('Fillet 0').*

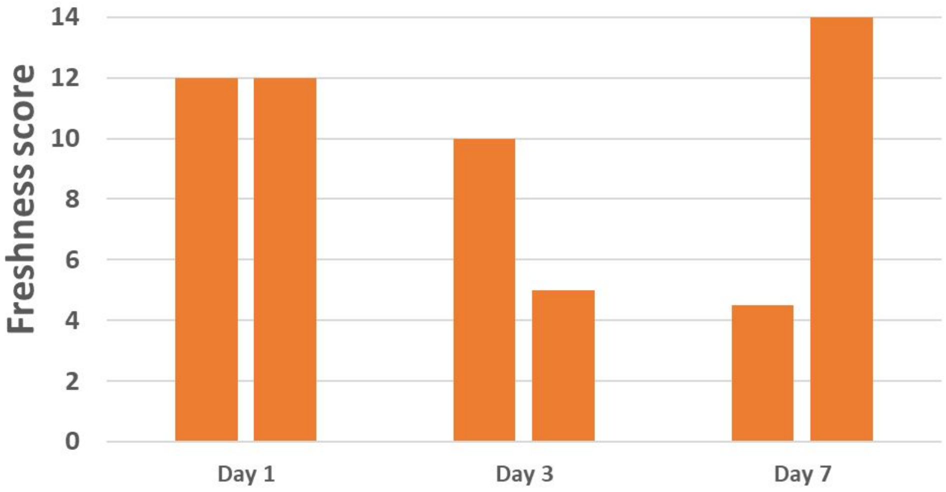

Fig.S3 *Organoleptic measurements on salmon fillet by six independent observers, for Days 1, 3 and 7, correlated with organoleptic chart freshness scores. Measurements on 'Fillet 1'.*

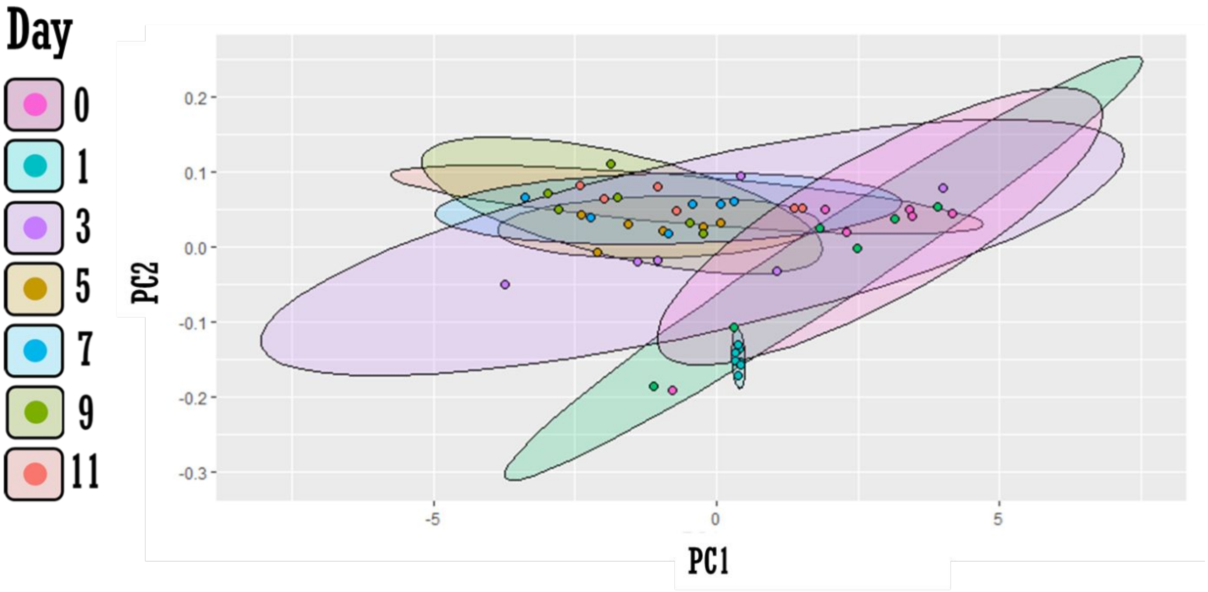

Fig.S4 *Principal Component Analysis for salmon fillet fluorescence data with variable selection (wavelengths: 453nm, 455nm, 457nm, 459nm to nearest integer value). Pooled/grouped PCA plot showing 'fresh', 'intermediate' and 'spoilt' categories in main text Figure4(d)(i) (color in print/online).*

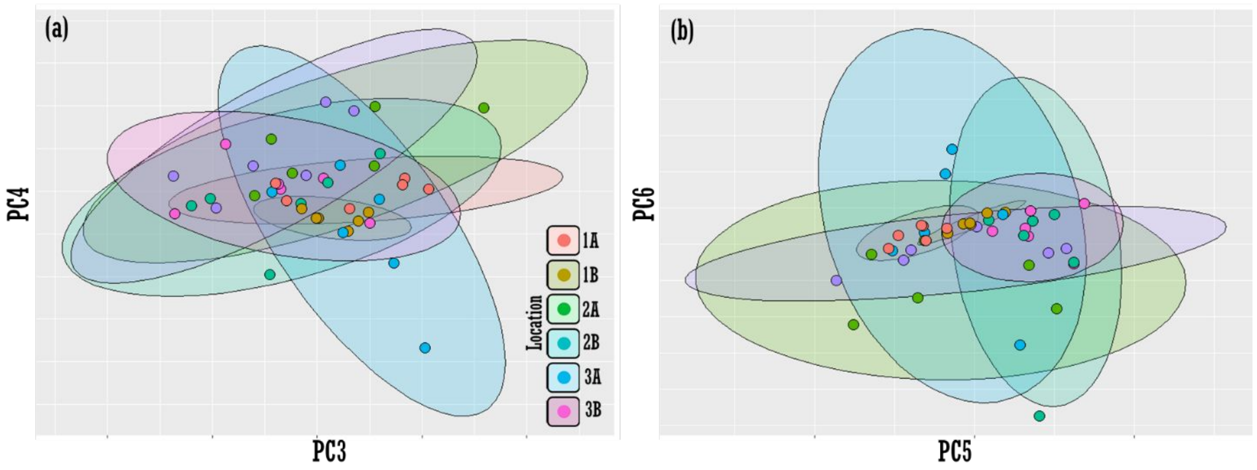

Fig.S5 *Principal Component Analysis for salmon fillet fluorescence data for higher PCs (a) PC3 vs. PC4 (b) PC5 vs. PC6. PC1 vs. PC2 in main text (color in print/online).*

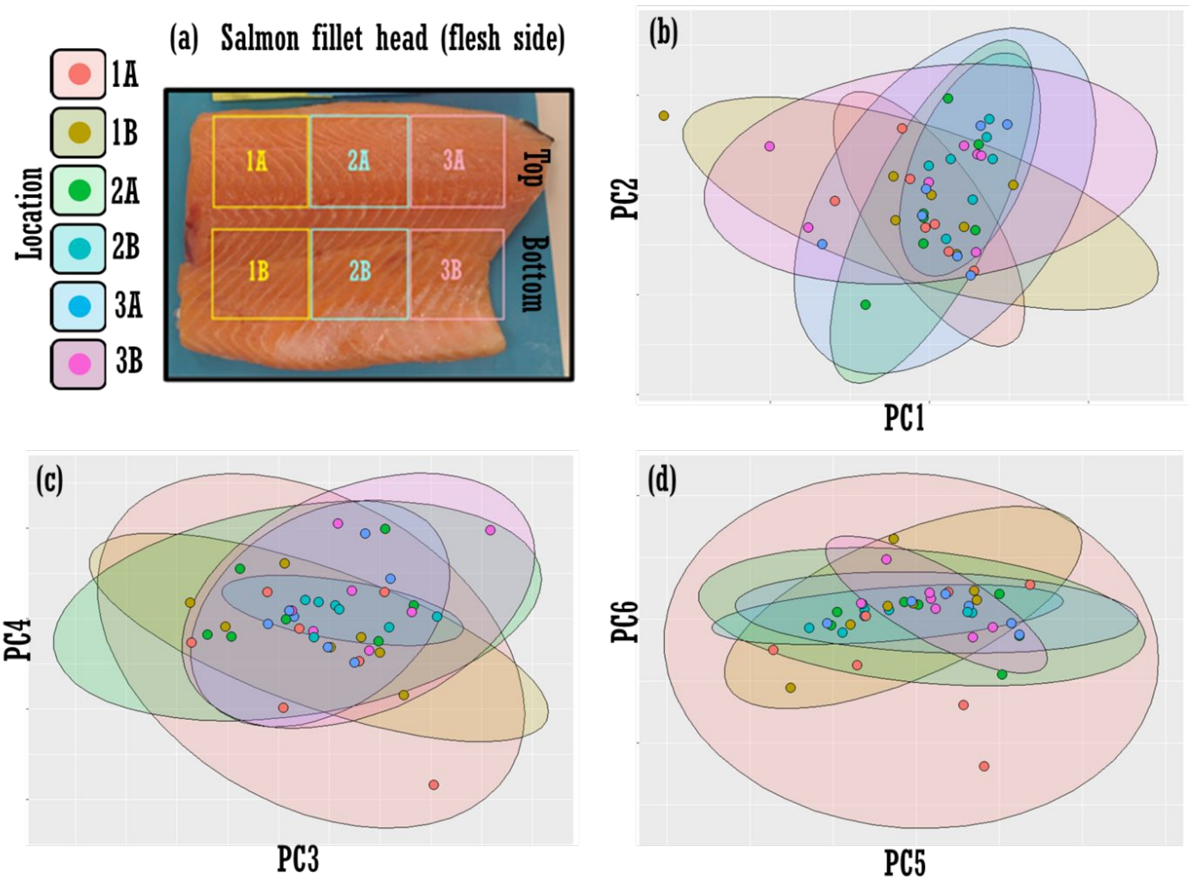

Fig.S6 *Principal Component Analysis by fillet location (a) Photo of salmon fillet and locations marked, (b) PC1 vs. PC2 (c) PC3 vs. PC4 (d) PC5 vs. PC6 (color in print/online).*

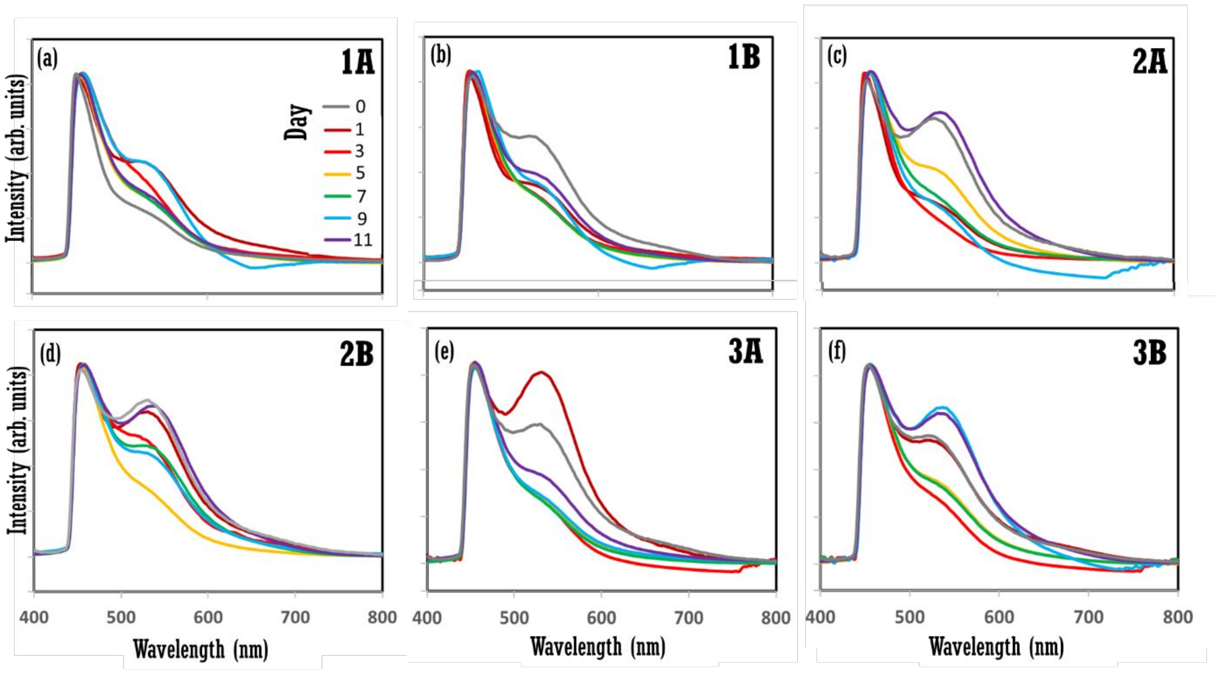

Fig.S7 *Overlaid scaled fluorescence data for all six fillet locations (a)-(f). Series show days. Baseline offsets corrected and spectra normalized to primary fluorescence peak (color in print/online).*

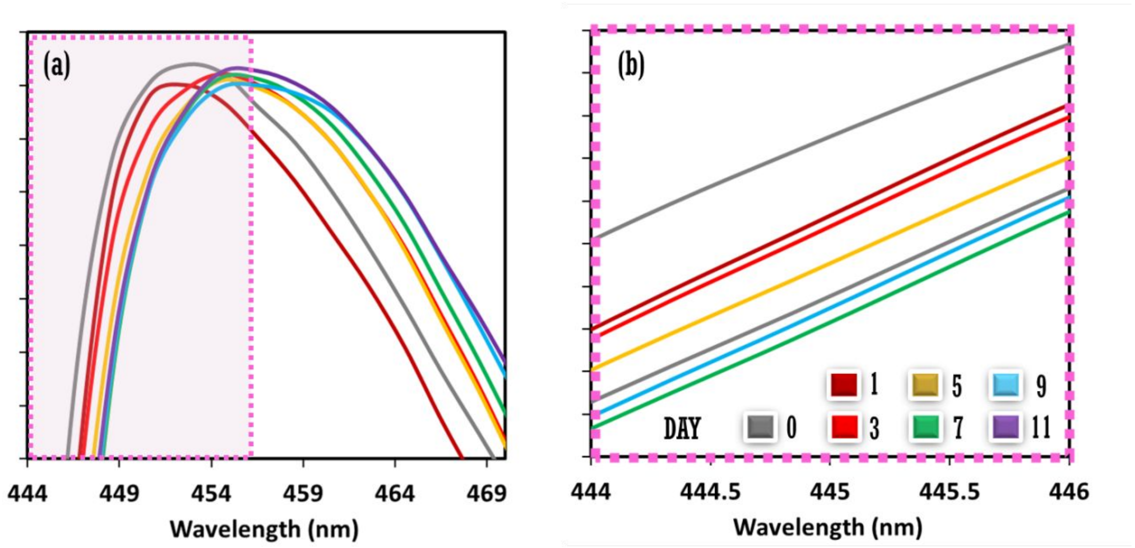

Fig.S8 *Fluorescence redshift (a) 444nm–469nm spectral window showing red shift as a function of measurement day, (b) Zoom of dashed region in (a) (444nm–446nm) (color in print/online).*

Fig.S9 *Extended variable Principal Component Analysis plot: 435nm–459nm (13 variables) with pooled categories as in Figure4(d)(i) (main text) (color in print/online).*

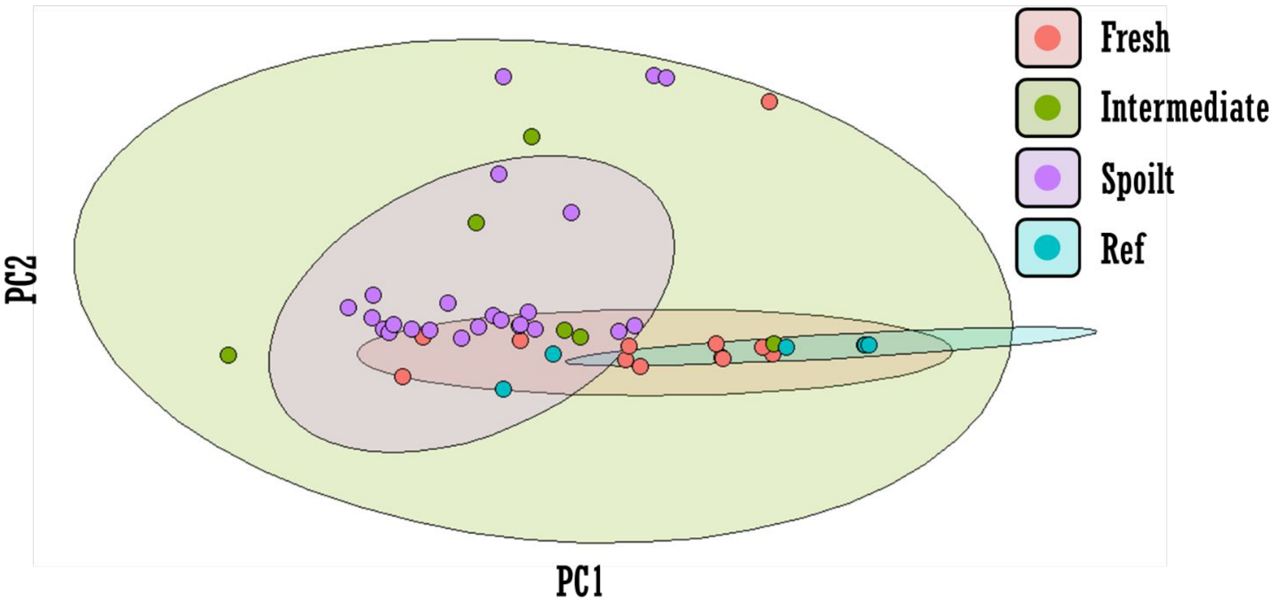

References

(1) Kashani Zadeh H, Hardy M, Sueker M, Li Y, Tzouchas A, MacKinnon N, Bearman G, Haughey S A, Akhbardeh A, Baek I, Hwang C, Qin J, Tabb A M, Hellberg R S, Ismail S, Reza H, Vasefi F, Kim M, Tavakolian K and Elliott C T 2023 Rapid Assessment of Fish Freshness for Multiple Supply-Chain Nodes Using Multi-Mode Spectroscopy and Fusion-Based Artificial Intelligence *Sensors* **23** 5149
